# Cadherin-13, a risk gene for ADHD and comorbid disorders, impacts GABAergic function in hippocampus and cognition

**DOI:** 10.1038/tp.2015.152

**Published:** 2015-10-13

**Authors:** O Rivero, M M Selten, S Sich, S Popp, L Bacmeister, E Amendola, M Negwer, D Schubert, F Proft, D Kiser, A G Schmitt, C Gross, S M Kolk, T Strekalova, D van den Hove, T J Resink, N Nadif Kasri, K P Lesch

**Affiliations:** 1 ADHD Clinical Research Unit, Division of Molecular Psychiatry, Department of Psychiatry, Psychosomatics and Psychotherapy, Center of Mental Health, University of Würzburg, Würzburg, Germany; 2 Department of Cognitive Neuroscience, Donders Institute for Brain, Cognition, and Behaviour, Radboud University Medical Center, Nijmegen, the Netherlands; 3 Department of Human Genetics, Donders Institute for Brain, Cognition, and Behaviour, Radboud University Medical Center, Nijmegen, the Netherlands; 4 Mouse Biology Unit, European Molecular Biology Laboratory, Monterotondo, Italy; 5 Department of Psychiatry, Psychosomatics and Psychotherapy, Center of Mental Health, University of Würzburg, Würzburg, Germany; 6 Department of Molecular Animal Physiology, Donders Institute for Brain, Cognition and Behaviour, Radboud University Nijmegen, Nijmegen, the Netherlands; 7 Department of Translational Neuroscience, School for Mental Health and Neuroscience, Maastricht University, Maastricht, the Netherlands; 8 Department of Biomedicine, Laboratory for Signal Transduction, University Hospital Basel, University of Basel, Basel, Switzerland

## Abstract

Cadherin-13 (CDH13), a unique glycosylphosphatidylinositol-anchored member of the cadherin family of cell adhesion molecules, has been identified as a risk gene for attention-deficit/hyperactivity disorder (ADHD) and various comorbid neurodevelopmental and psychiatric conditions, including depression, substance abuse, autism spectrum disorder and violent behavior, while the mechanism whereby CDH13 dysfunction influences pathogenesis of neuropsychiatric disorders remains elusive. Here we explored the potential role of CDH13 in the inhibitory modulation of brain activity by investigating synaptic function of GABAergic interneurons. Cellular and subcellular distribution of CDH13 was analyzed in the murine hippocampus and a mouse model with a targeted inactivation of *Cdh13* was generated to evaluate how CDH13 modulates synaptic activity of hippocampal interneurons and behavioral domains related to psychopathologic (endo)phenotypes. We show that CDH13 expression in the *cornu ammonis* (CA) region of the hippocampus is confined to distinct classes of interneurons. Specifically, CDH13 is expressed by numerous parvalbumin and somatostatin-expressing interneurons located in the stratum oriens, where it localizes to both the soma and the presynaptic compartment. *Cdh13*^−/−^ mice show an increase in basal inhibitory, but not excitatory, synaptic transmission in CA1 pyramidal neurons. Associated with these alterations in hippocampal function, *Cdh13*^−/−^ mice display deficits in learning and memory. Taken together, our results indicate that CDH13 is a negative regulator of inhibitory synapses in the hippocampus, and provide insights into how CDH13 dysfunction may contribute to the excitatory/inhibitory imbalance observed in neurodevelopmental disorders, such as ADHD and autism.

## Introduction

Brain development and plasticity are complex processes that require precise and specific interneuronal contacts to ensure the correct establishment of circuits at the cellular and systems level. To guarantee cellular and regional specification of synaptic contacts, the formation of neuronal networks is tightly controlled by a myriad of cell guidance and adhesion molecules, among which the cadherin superfamily of calcium-dependent cell adhesion molecules is of particular relevance.^1^ Furthermore, after development, cadherins regulate the function and plasticity of mature synapses by participating in synaptic vesicle dynamics or controlling the stability of synaptic receptors.^1^

Cadherin-13 (CDH13, also known as T-cadherin or H-cadherin) is an atypical member of the cadherin superfamily. It lacks both transmembrane and cytoplasmic domains and is linked to the plasma membrane via a glycosylphosphatidylinositol moiety.^2, 3, 4^ Genome-wide genetic approaches including linkage analyses, association studies and copy number variation studies have detected associations between the *CDH13* gene and various neurodevelopmental disorders. Notably, in several independent studies, *CDH13* has been associated with the risk for attention-deficit/hyperactivity disorder (ADHD) and frequent comorbid conditions including drug and alcohol abuse.^4, 5, 6, 7, 8, 9, 10, 11, 12, 13, 14, 15, 16, 17^ In addition, CDH13 was implicated in depression,^7, 18^ autism spectrum disorders,^19^ schizophrenia^20^ and bipolar disorder.^21^ Variations in *CDH13* have also been associated with the personality trait of extraversion^22^ and extremely violent behavior.^23^ The mechanism whereby CDH13 dysfunction contributes to the pathogenesis of neuropsychiatric conditions remains, however, unknown.

CDH13 is expressed in the developing and adult brain, as well as in the cardiovascular system, where it exerts its function via low-adhesive homophilic or heterophilic interactions to control cell migration, neurite outgrowth and axon guidance.^24, 25, 26, 27, 28, 29^ We therefore predicted that CDH13, as several other cell adhesion molecules, may have dual roles in the brain; specifically, as a guidance cue for migrating neurons and/or developing axons, and as a modulator of the establishment of specific synaptic contacts and function of mature synapses.^4^ The view that CDH13 may moderate synapse formation and plasticity is supported by a previous study,^30^ showing that *Cdh13* knockdown via RNA interference in hippocampal cultures resulted in a decrease in the density of both excitatory and inhibitory synapses.

Here, we focused on the hippocampus to test *in vivo* the hypothesis that involvement of *CDH13* in the susceptibility to neurodevelopmental/psychiatric disorders is associated with its potential role in the modulation of synaptic function. We investigated CDH13 expression and function in specific subtypes of hippocampal neurons *in vitro* and *in vivo.* Furthermore, we describe the electrophysiological and behavioral consequences of CDH13 deficiency in a mouse model of constitutive *Cdh13* inactivation. Finally, by linking alterations in hippocampal GABAergic synaptic transmission with cognitive deficits, we provide evidence that supports a critical role for CDH13 in the synaptic activity of the hippocampal GABAergic circuitry.

## Materials and methods

### Generation of a constitutive *Cdh13* knockout mouse line

A constitutive *Cdh13* knockout mouse (*Cdh13*^−/−^) was generated through site-specific DNA recombination by crossing *Cdh13*^loxP/loxP^ mice (C57Bl/6N background) with a constitutive Cre deleter line^31^ (Supplementary Figure S1 and Supplementary Table S1). The absence of CDH13 protein in *Cdh13*^−/−^ mice was verified by western blot and immunohistochemistry. Animal protocols have been approved by the boards of the University of Würzburg and the government of Lower Franconia, and performed according to the European Community guidelines for animal care and use. Adult mice (10–16 weeks) were used, if not indicated otherwise.

### Histological methods

#### *In situ* hybridization

Single chromogenic *in situ* hybridization to detect *Cdh13* messenger RNA (mRNA) was carried out on 16 μm coronal brain sections. Double-fluorescent *in situ* hybridization was performed to detect *Cdh13* (digoxigenin-labeled) and glutamic acid decarboxylase 1 (*Gad1*, fluorescein-labeled) mRNAs.

#### Immunofluorescence and colocalization assessment

Mice (*n*=3) were transcardially perfused, 40 μm free-floating serial coronal sections generated and co-stained for CDH13 plus one of the following interneuron markers: somatostatin (SOM), parvalbumin (PV), calbindin, neuropeptide Y, vasoactive intestinal peptide, neural nitric oxide synthase (nNOS) and calretinin. Some sections were triple-stained for CDH13, PV and SOM. Double immunofluorescence stainings were examined under an inverted epifluorescence microscope (Olympus, Hamburg, Germany). The numbers of single- and double-stained cells in single and merged images were counted using the ImageJ ‘Cell Counter' plugin.

#### Immunohistochemistry

For single CDH13 staining, 20 μm sections of perfusion-fixed brains on slides were incubated with primary antibody. N-Histofine Simple Stain Mouse MAX PO (G) (Nichirei Biosciences, Tokyo, Japan) and 3,3′-diaminobenzidine (Roche Diagnostics Deutschland, Mannheim, Germany) were used for detection.

For single staining of interneuronal markers, immersion-fixed brains (*n*=7–9 per genotype) were serially sectioned into 50 μm free-floating coronal sections. Sections were treated with heat-induced epitope retrieval, incubated with primary antibodies against SOM, PV and nNOS and detected using biotinylated secondary antibodies (Vector Laboratories, Burlingame, CA, USA), AB complex (Vector Laboratories) and 3,3′-diaminobenzidine (Roche Diagnostics Deutschland) development. Stained sections were then analyzed using Stereo Investigator v.11 (MBF Bioscience, Williston, VT, USA). PV-, SOM- and nNOS-positive cells in the stratum oriens (SO) were counted with the optical fractionator method.

#### Immunocytochemistry

Primary hippocampal neuron cultures were prepared from embryonic day 18 (E18) old rats as previously described^32^ and maintained in neurobasal media supplemented with B27, penicillin, streptomycin and l-glutamine (GIBCO-Invitrogen, Darmstadt, Germany). Fixed (paraformaldehyde) and permeabilized (Triton X-100) cultures were co-stained for CDH13 together with one of the following synaptic markers: vesicular glutamate transporter, vesicular GABA transporter, postsynaptic density protein 95 (PSD95), gephyrin and GABA-A receptor α1 subunit (GABA-Aα1). Double immunofluorescence stainings were examined under a Fluoview FV1000 confocal microscope (Olympus).

### RNA isolation and semiquantitative RT-PCR

Complementary deoxyribonucleic acid (cDNA) was synthesized using 1–2 μg total RNA from mouse brains (whole brain and hippocampus) of different ages. A 1:15 dilution of cDNA pool was used for semiquantitative RT-PCR analysis of *Cdh13* expression. The semiquantitative RT-PCR data were analyzed using comparative quantification. Peptidyl prolyl isomerase (*Ppia*) and β-actin (*Actb*) served as reference genes for normalization of *Cdh13* Q-values.

### Electrophysiology

Electrophysiological experiments were conducted on horizontal slices (350-μm thick) from the ventral hippocampus of postnatal day (P) 20–22 mice of both genders. Miniature inhibitory postsynaptic currents (mIPSCs) were recorded in the presence of Tetrodotoxin (1 μm), 6-cyano-7-nitroquinoxaline-2,3-dione (CNQX, 5 μm) and D-(-)-2-amino-5-phosphonopentanoic acid (D-APV, 100 μm). Miniature excitatory postsynaptic currents were recorded in the presence of Tetrodotoxin and Picrotoxin (100 μm). Paired pulse ratio was recorded in the presence of 6-cyano-7-nitroquinoxaline-2,3-dione, D-(-)-2-amino-5-phosphonopentanoic acid, 4 mm CaCl_2_ and 4 mm MgCl_2_. Traces were recorded using a Multiclamp 700B amplifier (Molecular Devices, Wokingham, UK). Miniature recordings were analyzed using Mini Analysis Program (Synaptosoft, Decatur, GA, USA). Other traces were analyzed using Clampfit 10.2. Intrinsic cell parameters were recorded in current clamp using Signal (CED, Cambridge, UK) and traces were analyzed using Stimfit v0.13 following the parameter definitions used in Tricoire *et al.*^33^

### Morphological reconstructions

Brain slices with biocytin-filled neurons were processed following a modified staining protocol based on Marx *et al.*^34^ Stained sections were imaged and somata, apical and basal dendrites of selected pyramidal neurons were reconstructed in Neurolucida v10 (MBF Bioscience). Reconstructions were analyzed in NeuroExplorer (MBF Bioscience) for intrinsic parameters and Sholl analysis.

### Behavioral assessment

Four cohorts of adult male *Cdh13*^+/+^ (*n*=7–10), *Cdh13*^+/−^ (*n*=8–10) and *Cdh13*^−/−^ (*n*=7–10) mice were subjected to behavioral tests to assess anxiety-like behavior and conditioned fear responses, depression-like behavior, social interaction, locomotor activity, attention, impulsivity and cognitive flexibility. Stress hormone levels were measured from fecal samples and central blood.

### Statistical analysis

Genotype effects were analyzed by univariate analysis of variance (two-sided) and, in cases of significance (*P*<0.05), *post hoc* comparisons using Bonferroni correction for multiple testing. In cases of non-normality and/or unequal variances, non-parametric statistical analysis using the Kruskal–Wallis test was performed instead of parametric analysis of variance. For those behavioral tests with repeated measures, a mixed analysis of variance was used, with genotype as between-subject factor and time or testing phase as repeated measure. Data analysis was performed using Prism 6.04 (GraphPad Software, La Jolla, CA, USA) or SPSS Statistics 21 (IBM, Armonk, NY, USA). A preliminary analysis revealed that the statistical power of our experimental setups to detect genotype-dependent differences with a medium effect size was of at least 0.8.

Detailed descriptions of all the methods are provided in the Supplementary Information.

## Results

### CDH13 is located in specific subtypes of hippocampal interneurons

**Figure 1:**
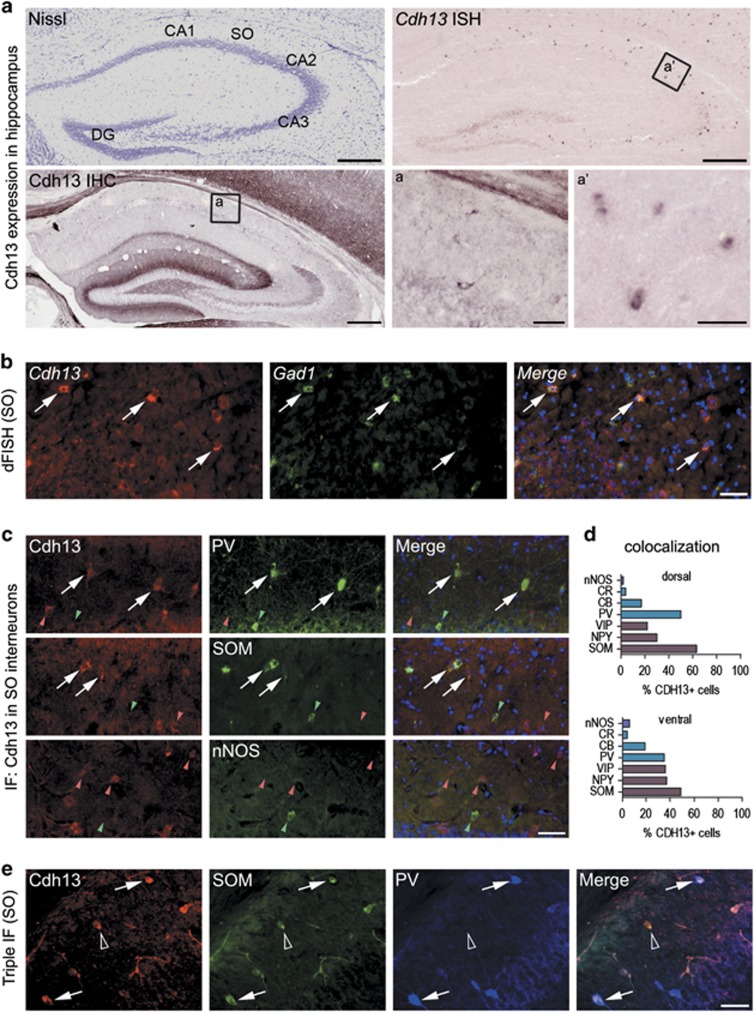
CDH13 expression in murine hippocampus. (**a**) CDH13 protein and mRNA expression in the hippocampus of the adult mouse, CDH13 protein and mRNA are present in cell somata of the stratum oriens (SO; a,a'). (ISH picture was shown as preliminary data in a review article by Rivero *et al.*;^4^ reproduced with permission from Elsevier). (**b**) Double FISH: *Cdh13* is localized in *Gad1* mRNA-positive cells of the SO (white arrows: colocalization; green arrows: only *Gad1+*). (**c**) IF images: colocalization study of CDH13 and parvalbumin/somatostatin/neural nitric oxide synthase (white arrows: colocalization, green arrowhead: single stained cell for PV/SOM/nNOS, red arrowhead: single stained cell for CDH13). (**d**) Colocalization of CDH13 with neurochemical markers of GABAergic interneurons in the SO of the dorsal (upper panel) and ventral (lower panel) hippocampus. A higher degree of colocalization is observed in the dorsal part of the hippocampus. *n*=3; 100%=CDH13+ cells; percentages calculated from median (see Supplementary Figure S2 for details). (**e**) Triple IF: CDH13 is colocalized with PV and SOM in some but not in all cells of the SO (white arrow: triple localization, empty arrowhead: double localization). Scale bars, 300 μm in **a**, 50 μm in a/a', **b**, **c** and **e**. CB, calbindin; CR, calretinin; DG, dentate gyrus; FISH, fluorescent *in situ* hybridization; mRNA, messenger RNA; nNOS, neural nitric oxide synthase; NPY, neuropeptide Y; PV, parvalbumin; SOM, somatostatin; VIP, vasoactive intestinal peptide.

We detected *Cdh13* mRNA in subregions and distinct cells of the hippocampus (Figure 1). In particular, the highest cellular expression was detected in the SO of the *cornu ammonis* (CA) region, whereas weaker labeling was observed in scattered cells of the CA3 pyramidal layer, as well as the hilus and the granule cell layer of the dentate gyrus. These *in situ* hybridization findings were complemented with an immunohistochemical analysis of CDH13 protein expression using an anti-CDH13 antibody which we validated for specificity using *Cdh13* null mutant mice (Supplementary Figure S1). Of note, intense cellular immunoreactivity was observed in cell bodies located in the SO, in a pattern which closely resembled *Cdh13* mRNA expression. In addition, CDH13 protein was also distributed in the hippocampus in a characteristic laminar pattern (Figure 1a), with the strongest staining in the stratum lucidum of CA3 and stratum lacunosum-moleculare, in the molecular layer and hilus of the dentate gyrus, as well as along the mossy fiber projections.

The unique and distinct cellular pattern of CDH13 labeling in the hippocampal SO prompted us to ascertain the nature of these CDH13-positive cells. Their sparse distribution suggested a GABAergic nature, as it is known that the bodies of a considerable number of GABAergic inhibitory interneurons are located in this deep layer of the hippocampus.^35^ As expected, a double-fluorescent *in situ* hybridization approach using probes for *Cdh13* and the interneuron marker *Gad1* showed that most of the *Cdh13*-positive somata of the SO were indeed interneurons (Figure 1b).

Hippocampal interneurons are a heterogeneous group of cells that differ in their morphological and electrophysiological properties.^33^ To better identify the nature of the CDH13-immunoreactive interneurons detected in the SO, we performed double-immunofluorescence experiments to label CDH13 with each of seven neurochemical markers (PV, calbindin, calretinin, SOM, neuropeptide Y, vasoactive intestinal peptide and nNOS) characteristically expressed in different subpopulations of hippocampal interneurons (Figures 1c and d). Initial qualitative assessment revealed that the extent of coexpression with CDH13 substantially varied depending on the analyzed interneuron marker (Figure 1c). Next, a stereological approach to precisely quantify the degree of coexpression (Figure 1d and Supplementary Figure S2a) was used. The highest levels of coexpression were found with SOM (63% of CDH13-immunoreactive cells were also positive for SOM in the dorsal hippocampus, 49% in the ventral hippocampus) and PV (with 50% of CDH13-immunoreactive cells being positive for PV in the dorsal hippocampus, 35% in the ventral part). In contrast, very low levels of coexpression were found with nNOS (2–6%) and calretinin (4%). In addition, the percentages of interneurons of each subpopulation that were positive for CDH13 were calculated (Supplementary Figure S2a). In this case, CDH13 was most frequently found in PV- (66% dorsal, 31% ventral), SOM- (55% dorsal, 39% ventral) and neuropeptide Y- (47% dorsal, 35% ventral) immunoreactive cells of the SO.

The above data indicate that, in the SO, CDH13 is most frequently found in PV- and SOM-positive cells. PV and SOM are thought to be representative of two interneuronal subpopulations that are distinct or have very little overlap. There is no coexpression of PV and SOM in the cerebral cortex,^36^ whereas there is a small degree of overlap in the hippocampus.^33^ Notably, we show via triple PV-SOM-CDH13 staining that some, but not all, of the CDH13-immunoreactive cells of the SO were positive for both SOM and PV (Figure 1e).

### CDH13 is located at inhibitory synapses

**Figure 2:**
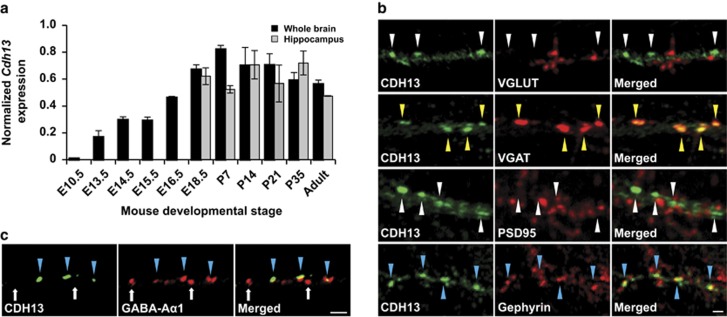
Temporal expression pattern and subcellular localization of CDH13. (**a**) Developmental timeline of *Cdh13* mRNA expression for whole brain (black bars) and hippocampus (gray bars; mean±s.e.m.; *n*=2 for hippocampus E18.5, P7, P21 and adult, for all other time points *n*=3). (**b**) Immunofluorescence staining of cultured hippocampal neurons for CDH13 (green) and the synaptic markers VGLUT, VGAT, PSD95 or Gephyrin (red). CDH13-positive puncta are indicated by the triangles, colored yellow for colocalized foci (VGAT) and blue for juxtaposed foci (Gephyrin). CDH13 localizes at the presynaptic side of inhibitory synapses. Scale bar, 1 μm. (**c**) Immunofluorescence staining of cultured hippocampal neurons for CDH13 (green) and GABA-A receptor α1 subunit (GABA-Aα1, red). CDH13-positive puncta are indicated by the triangles, colored blue for juxtaposition with GABA-Aα1-positive puncta. White arrows indicate puncta that are CDH13-negative and GABA-Aα1-positive. Scale bar, 1 μm. mRNA, messenger RNA; VGAT, vesicular GABA transporter; VGLUT, vesicular glutamate transporter.

To gain further insight into the function of CDH13 in the brain, we assessed its temporal expression pattern in whole brain as well as in hippocampus (Figure 2a). *Cdh13* mRNA was detected from E13.5 onwards and increased to reach a peak at P7 to P21 for whole brain, as well as P14 for hippocampus, times of period that coincide with synapse formation.^30, 37^ Although previous studies showed that ectopically expressed CDH13 localized to synapses in hippocampal primary cultures,^30^ we explored endogenous labeling at the synapse in more detail. Hippocampal neurons at 3 days *in vitro* were transfected with a myc-tagged *Cdh13.*^30^ Co-staining for CDH13 and MYC at 14 days *in vitro* showed an exactly overlapping punctated pattern and confirmed antibody specificity for subsequent co-immunofluorescence analysis (Supplementary Figure S3). To determine the specific synaptic localization of endogenous CDH13, we co-stained hippocampal neurons at 14 days *in vitro* for CDH13 and the presynaptic markers vesicular glutamate transporter (VGLUT) or vesicular GABA transporter (VGAT) (excitatory and inhibitory, respectively) or the postsynaptic markers PSD95 or gephyrin (excitatory and inhibitory, respectively; Figure 2b). CDH13 colocalized exactly with the presynaptic marker VGAT, but not VGLUT. At the postsynaptic side, CDH13 showed juxtaposed labeling with gephyrin, but not with PSD95. As CDH13 is coexpressed in a high proportion of PV-positive cells (Supplementary Figure S2a), we investigated whether CDH13 colocalized with the GABA-A subunit primarily found in synapses formed by PV-positive cells, GABA-Aα1 (Figure 2c). Indeed, CDH13 puncta were found juxtaposed to GABA-Aα1 puncta, consistent with a presynaptic (CDH13) and postsynaptic (GABA-Aα1) localization. Conversely, not all GABA-Aα1-positive puncta were positive for CDH13, indicating that these puncta originate from another subpopulation of interneurons that is CDH13-negative. Together with our finding that CDH13 is expressed in inhibitory neurons in the SO, these data provide evidence that, in hippocampal neurons, CDH13 localizes at the presynaptic compartment of inhibitory synapses.

### *Cdh13* knockout mice display increased inhibitory synaptic transmission

To investigate the functional consequences of CDH13 deficiency for the hippocampal inhibitory network, we generated a constitutive *Cdh13* knockout mouse line (Supplementary Figure S1). In agreement with a previously reported mouse model of constitutive CDH13 deficiency,^38^ *Cdh13* null mutant (*Cdh13*^−/−^) mice exhibited normal life expectancy and breeding capacity; they did not present any gross morphological abnormalities of the brain (Supplementary Figure S4). Weight gain, daily food and water intake, as well as stress hormone levels were not affected by genotype (Supplementary Figure S5).

**Figure 3:**
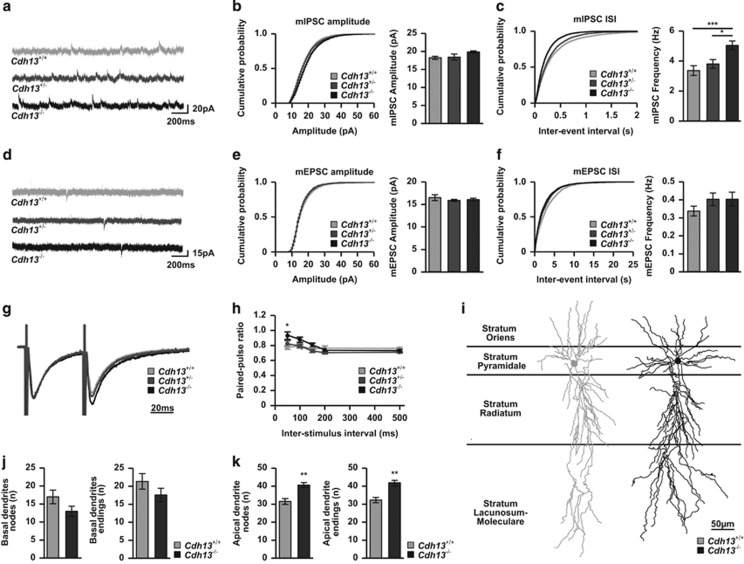
Hippocampal CA1 neurons from *Cdh13* null mutant mice show increased inhibitory synaptic input and morphological changes. (**a**) Representative traces of miniature inhibitory postsynaptic currents (mIPSCs) recorded from CA1 pyramidal cells from acute brain slices from *Cdh13*^*+/+*^, *Cdh13*^+/−^ and *Cdh13*^−/−^ mice. (**b** and **c**) Cumulative distributions of mIPSC amplitudes (**b**) and inter-event intervals (IEI; **c**) from *Cdh13*^+/+^ (*n*=19/3), *Cdh13*^+/−^ (*n*=18/3) and *Cdh13*^−/−^ (*n*=23/4) hippocampal CA1 neurons. (**d**) Representative traces of miniature excitatory postsynaptic currents (mEPSCs) recorded from CA1 pyramidal cells from acute brain slices of *Cdh13*^*+/+*^, *Cdh13*^+/−^ and *Cdh13*^−/−^ mice. (**e** and **f**) Cumulative distributions of mIPSC amplitudes (**e**) and IEI (**f**) from *Cdh13*^*+/+*^ (*n*=12/3), *Cdh13*^+/−^ (*n*=13/3) and *Cdh13*^−/−^ (*n*=14/3) hippocampal CA1 neurons. (**g**) Representative overlaid traces of two IPSCs evoked by stimuli delivered at an inter-stimulus interval (ISI) of 50 ms. (**h**) Quantification of paired pulse ratio following stimulation in SO for *Cdh13*^*+/+*^ (*n*=15/4), *Cdh13*^+/−^ (*n*=15/3) and *Cdh13*^−/−^ (*n*=15/3). (**i**) Representative reconstructions of hippocampal CA1 neurons from *Cdh13*^*+/+*^ and *Cdh13*^−/−^ mice. (**j** and **k**) Quantification of (**j**) basal dendritic and (**k**) apical dendritic nodes and endings for *Cdh13*^*+/+*^ (*n*=6) and *Cdh13*^−/−^ (*n*=7). Bar graphs are presented as mean±s.e.m. *P<0.05; **P<0.01; ***P<0.001. *n* is shown as number of cells/number of animals.

As CDH13 is localized at the presynaptic compartment of inhibitory synapses, we assessed basal inhibitory synaptic transmission by recording mIPSCs from CA1 pyramidal cells in acute slices from P20-22 *Cdh13*^+/+^, *Cdh13*^+/−^ and *Cdh13*^−/−^ mice (Figures 3a to c). We found an increased mIPSC frequency in the *Cdh13*^−/−^ mice compared with *Cdh13*^+/+^ (*Cdh13*^*+/+*^: 3.29±0.47 Hz, *Cdh13*^−/−^: 4.93±0.29 Hz, *P*<0.001) and *Cdh13*^+/−^ (3.73±0.29 Hz, *P*<0.05), but no change in mIPSC amplitude (*Cdh13*^*+/+*^: 18.25±0.42 pA, *Cdh13*^−/−^: 19.31±0.36 pA, *P*=0.07). Consistent with exclusive localization of CDH13 at inhibitory synapses, miniature excitatory postsynaptic currents were unchanged in both amplitude (*Cdh13*^*+/+*^: 16.43±0.67 pA, *Cdh13*^+/−^: 15.85±0.24 pA, *Cdh13*^−/−^: 16.00±0.32 pA, *P*>0.05) and frequency (*Cdh13*^*+/+*^: 0.34±0.03 Hz, *Cdh13*^+/−^: 0.40±0.03 Hz, *Cdh13*^−/−^: 0.40±0.04 Hz, *P*>0.05; Figures 3d to f). As our approach was constitutive rather than interneuron-specific *Cdh13* inactivation, we aimed to exclude the possibility that knocking out *Cdh13* in CA1 pyramidal cells has any effects on synaptic transmission. To do this, we prepared organotypic hippocampal slices from wild-type rats and injected a lentivirus carrying a shRNA against *Cdh13* previously validated by Paradis *et al.*^30^ into the pyramidal cell layer of CA1 and then recorded simultaneously from an infected and a non-infected pyramidal neuron and compared the response to different stimulation locations (Supplementary Figures S6a, e and i). We found no difference in inhibitory input from SO (Supplementary Figures S6b to d) or stratum radiatum (Supplementary Figures S6f and g). Also, excitatory input from stimulation of the Schaffer collaterals was unaltered for both AMPA (Supplementary Figures S6j and k) and NMDA (Supplementary Figures S6l and m) receptor-mediated input. We therefore conclude that it is unlikely that the constitutive nature of our knockout model affects excitatory pyramidal cells directly, and the observed increase in mIPSC frequency is more likely caused by the inactivation of *Cdh13* in presynaptic inhibitory neurons.

Generally, changes in frequency are explained as either a change in presynaptic release probability, or as a change in synapse number. To enable distinction between both possibilities, we recorded the paired pulse ratio, a measure of the release probability, of inhibitory synapses following stimulation in SO at different inter-stimulus intervals. We found an increased paired pulse ratio in *Cdh13*^−/−^ compared to *Cdh13*^*+/+*^ mice at the 50 ms inter-stimulus interval (ISI) only (ISI 50 ms: *Cdh13*^*+/+*^: 0.77±0.03, *Cdh13*^−/−^: 0.93±0.05, *P*<0.05, Figures 3g and h). An increased paired pulse ratio is associated with a decreased release probability. Therefore, the underlying cause for increased mIPSC frequency in *Cdh13*^−/−^ mice is unlikely to be a change in release probability, but rather a change in the number of inhibitory synapses formed onto CA1 pyramidal cells.

To investigate whether the changes in inhibitory synapses could be due to altered development of interneuron subtypes we analyzed the density of PV-, SOM- and nNOS-positive interneurons of the SO in adult *Cdh13*^−/−^, *Cdh13*^+/−^ and *Cdh13*^+/+^ mice. We found no effect of CDH13 deficiency on the densities of nNOS-positive (which rarely colocalize with CDH13), PV- or SOM-positive interneurons (*P*>0.05, Supplementary Figure S2b). These results exclude that the enhanced inhibitory synaptic input on CA1 neurons in *Cdh13*^−/−^ mice is owing to an increased amount of interneurons in the SO. Furthermore, no differences in the hippocampal levels of transmitters (glutamate, GABA, dopamine, serotonin and norepinephrine) or their metabolites were found between genotypes (Supplementary Figure S7).

Next, we assessed the general dendritic morphology of CA1 pyramidal cells. To this end, CA1 pyramidal cells were loaded with biocytin for morphological reconstruction of apical and basal dendrites (Figure 3i). The basal dendrites were not different between *Cdh13*^*+/+*^ and *Cdh13*^−/−^ animals (Figure 3j). The apical dendrites, on the other hand, were found to be significantly more complex in *Cdh13*^−/−^ animals at the level of dendritic nodes and length (nodes: *Cdh13*^*+/+*^: 31.5±1.6, *Cdh13*^−/−^: 40.6±1.4, *P*<0.01; endings *Cdh13*^*+/+*^: 32.3±1.5, *Cdh13*^−/−^: 41.9±1.7, *P*<0.01; length *Cdh13*^*+/+*^: 3041.5±436.1 μm, *Cdh13*^−/−^: 4521.4±478.9 μm, *P*<0.05; Figure 3k, Supplementary Table S2). However, analysis of the space occupied by the dendrites (Convex Hull analysis, Supplementary Table S2) showed no difference between genotypes, indicating that the apical dendrite stays within the same envelope, but is more branched in *Cdh13*^−/−^ animals. Intrinsic electrophysiological properties, such as membrane potential, input resistance and action potential properties, were identical between *Cdh13*^*+/+*^ and *Cdh13*^−/−^ animals (Supplementary Table S3). Together, these data indicate that CDH13 is a negative regulator of inhibitory synapse function. Thus, loss of function of *Cdh13* could lead to a distorted excitation/inhibition balance within the hippocampus.

### Behavioral alterations of *Cdh13* knockout mice correlate with hippocampal dysfunction and ADHD phenotypes

**Figure 4:**
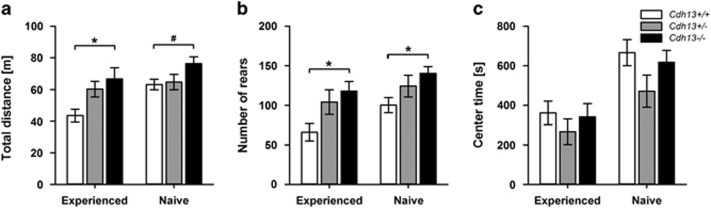
*Cdh13*^−/−^ mice display novelty-induced hyperlocomotion but unaltered anxiety-like behavior in the open field. (**a**) Total distance traveled and (**b**) number of rears as measures of locomotor activity. (**c**) Time spent in the center of the open field as measure of anxiety-like behavior. Experienced: *n*=7–8 per genotype, naive: *n*=10 per genotype. Data are presented as mean±s.e.m. ^#^*P*<0.1, *P<0.05.

We assessed the effects of CDH13 deficiency on different behavioral domains. The analysis revealed no significant genotype effect on any variable measured to assess anxiety- and depression-like behavior as well as social interaction (Supplementary Figures S8–S10). We also tested locomotor activity in an open field for 30 min. In a cohort of mice with prior test experience, we detected a significant genotype effect for distance traveled (*P=*0.023, Figure 4a) and number of rears (*P*=0.039, Figure 4b), indicating that *Cdh13*^−/−^ mice displayed higher locomotor activity than *Cdh13*^+/+^ mice. These findings were replicated in a cohort of naive mice (distance: *P*=0.069; rears: *P*=0.047). Center time, a measure of anxiety-like behavior, was not affected by genotype in either test-experienced or naive cohorts (Figure 4c).

**Figure 5:**
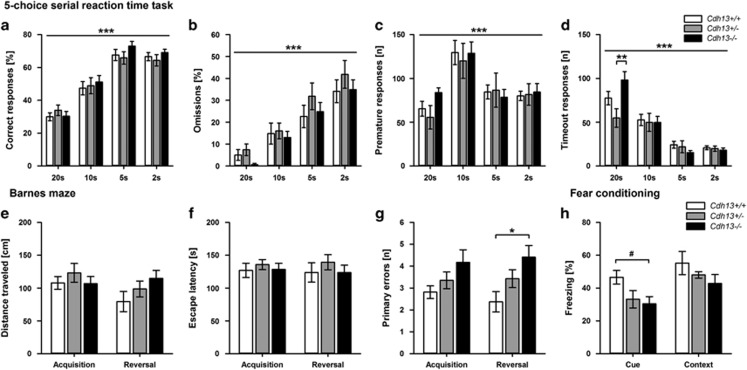
*Cdh13*^−/−^ mice exhibit normal attentional performance in the 5-CSRTT but impaired spatial learning in the Barnes maze and deficits in cued fear memory following delay fear conditioning. (**a**) % number of correct responses, (**b**) % number of omission errors, (**c**) number of premature responses and (**d**) number of timeout responses at different stimulus durations in the 5-CSRTT (*n*=10 per genotype). (**e**) Total distance traveled, (**f**) escape latency and (**g**) number of primary errors during acquisition and reversal phase of the Barnes maze (*n*=7–10 per genotype). (**h**) Freezing (%) duration during presentation of the auditory cue and the original conditioning context following delay fear conditioning (*n*=9–10 per genotype). Data are presented as mean±s.e.m. ^#^*P*<0.1, *P<0.05, **P<0.01, ***P<0.001. 5-CSRTT, five-choice serial reaction time task.

In the five-choice serial reaction time task (5-CSRTT), which evaluates attention and impulsivity, acquisition and performance of the task was similar in all genotypes, as indicated by gradually increasing accuracy, number of omission errors and pellets eaten, indicating unaltered attention in *Cdh13*^−/−^ mice, as well as decreasing number of premature and timeout responses and faster response latencies, findings that support no genotype-dependent differences in impulsivity (Figure 5 and Supplementary Figure S11; session effects: all *P*<0.001). No significant genotype effects on any variable measured in the 5-CSRTT were detected. However, there was a session × genotype interaction for the number of timeout responses (Figures 5d, *P*=0.005), showing that during the first training session with longer stimulus duration of 20 s, *Cdh13*^−/−^ mice made significantly more nose-pokes after an incorrect response, but only when compared with *Cdh13*^+/−^ mice.

The assessment of hippocampus-dependent visuospatial memory in the Barnes maze showed that all mice learned to find the hidden escape box in both acquisition and reversal, as indicated by gradually decreasing distances, escape latencies and primary errors throughout the successive trials (significant session effects: all *P*<0.001; data not shown). There were no session × genotype interactions, thus data were pooled across sessions to assess genotype effects. Distance traveled, escape latency and primary errors (Figures 5e to g) did not differ between genotypes during acquisition. However, during reversal, where the escape box is located in a different position, *Cdh13*^−/−^ mice made significantly more primary errors than *Cdh13*^+/+^ mice (*P*=0.027), indicating impaired cognitive flexibility.

During fear conditioning, all genotypes displayed similar responses to footshock as well as significantly increased post-shock freezing (time effect: *P<*0.001 compared with pre-shock), reflecting unaltered pain sensitivity and normal acquisition of conditioned fear in *Cdh13*^−/−^ mice (Supplementary Figures S12a and b). Twenty-four hours cued fear memory was significantly impaired in *Cdh13*^−/−^ mice compared with *Cdh13*^+/+^ mice, as indicated by decreased freezing (genotype main effect: *P=*0.047, Figure 5h) and increased activity (*P=*0.081, Supplementary Figure S12c) during tone presentation. Similarly, in the 48-h contextual fear memory test, *Cdh13*^−/−^ mice tended to be more active than *Cdh13*^+/+^ mice (*P=*0.094), although freezing duration did not significantly differ between genotypes when exposed to the original conditioning chamber.

In summary, CDH13 deficiency impacts locomotor activity as well as learning and memory, two behavioral domains frequently associated with ADHD and related neurodevelopmental disorders.

## Discussion

Variation in *CDH13* has been associated with a wide range of neurodevelopmental and psychiatric disorders, ranging from ADHD to autism spectrum disorders, among others.^4^ Although functional single-nucleotide polymorphisms/variants or haplotypes in *CDH13* have yet to be linked to neurodevelopmental/psychiatric disorders, several copy number variant studies revealed deletions in ADHD and autism spectrum disorder patients that may disrupt CDH13 expression and/or function.^19, 39, 40^ Our findings revealed that inhibitory interneurons are the primary locus of CDH13 in the murine hippocampus, and that CDH13 deficiency affects hippocampal inhibitory function and several behavioral domains that are fundamentally altered across various neuropsychiatric phenotypes, thus contributing to shed light on the role of CDH13 in the etiopathogenesis of neurodevelopmental and psychiatric disorders.

We confirmed that in the hippocampal CA region, CDH13 is predominantly expressed in *Gad1* mRNA-positive cells, with preference for PV- and SOM-positive interneurons, thus allocating CDH13-related functions to several GABAergic interneuron subpopulations: axo-axonic cells, basket cells, bistratified cells, and also oriens lacunosum-moleculare or hippocampal-septal projection cells.^33, 41^ Alterations in PV interneurons have previously been linked to various neuropsychiatric and neurodevelopmental disorders as well as related animal models.^42, 43^ Furthermore, SOM-positive interneurons have been implicated in the acquisition of contextual fear memories and the regulation of hippocampal synaptic plasticity.^44^

Along with several other members of the cadherin superfamily that have been demonstrated to regulate interneuron development and hippocampal synaptic function,^45, 46, 47, 48^ CDH13 expression in the hippocampal CA region suggests a function in interneurons, particularly at the inhibitory synapse. Furthermore, we confirmed that *Cdh13* mRNA expression reaches its peak in the developing brain of the mouse between P7 and P21, consistent with recent observations in lysates from rat cortex,^29^ thus supporting the view that CDH13 expression increments coincide with peaking synaptogenesis in the neocortex.^30, 37^ Our investigations also show that CA1 neurons from *Cdh13*^−/−^ mice receive increased inhibitory input, whereas no changes were found in excitatory input, which is likely to result in an excitatory–inhibitory imbalance. This notion supports the view of CDH13 being a critical regulator of inhibitory function in the hippocampus consistent with its localization in the GABAergic presynaptic compartment. The fine-tuning of hippocampal circuitry depends greatly on the precise activity of the inhibitory system to control hyperexcitability in the hippocampus, which displays the lowest seizure threshold of the entire brain.^49^ However, alterations associated with an altered inhibitory function are pathophysiologically beyond epilepsy. Multiple lines of evidence support the role of the inhibitory GABAergic system in the pathogenesis of psychiatric disorders.^42, 50^ Accordingly, disturbances of the excitatory–inhibitory balance lead to deficits in information processing and signal gating, two key processes involved in attentional and executive control.^42, 51, 52, 53, 54^ Candidate risk genes studies further support the view that alterations in the inhibitory system likely account for a spectrum of neuropsychiatric manifestations, including schizophrenia, autism spectrum disorders and ADHD.^42, 55, 56, 57, 58^ As an example, ErbB4 receptor tyrosine kinase, which is almost exclusively expressed in interneurons, has strongly been implicated in schizophrenia.^56, 59^ In the hippocampus, ErbB4 is located at the postsynaptic compartment of glutamatergic synapses of PV-positive interneurons^60^ and has a critical role in the regulation of long-term potentiation at the SC-CA1 synapse via these interneurons.^61, 62^ In the thalamic reticular nucleus, ErbB4 is mainly expressed in SOM-positive interneurons and is important for sensory selection.^51^ Of note, we also observed strong expression of *Cdh13* in thalamic reticular nucleus neurons.^4^ In autism, the different isoforms of the cell adhesion proteins neurexins and neuroligins have both been shown to have critical roles in inhibitory synapse function. Of particular relevance is the observation that neuroligins 2 (ref. 63) and 3 (ref. 64) have distinct functions depending on the interneurons with which they form a synapse. Analogous to the neuroligins, and as we show that CDH13 is expressed in both PV- and SOM-immunoreactive cells, CDH13 might have distinct functions depending on the interneurons in which it is expressed.

Although the concentrations of GABA in hippocampus appear to be unchanged in *Cdh13*^−/−^ mice, CDH13 deficiency may have effects on the modulation of inhibitory synaptic activity and synapse turnover. Our electrophysiological findings support the view that CDH13 may act as a synapse destabilizer. Therefore, in the absence of CDH13, stability of GABAergic synapses might be augmented owing to a reduced synapse turnover, producing an increase in inhibitory synapse density, consistent with the observed increase in inhibitory synaptic transmission. The role of cadherins in synapse (de)stabilization and plasticity has been well documented.^1, 65, 66^ The mechanism whereby CDH13 would participate in synapse (de)stabilization might involve heterophilic interactions in *trans* with elements of the postsynaptic compartment and/or also interactions in *cis* with presynaptic transmembrane molecules in close proximity. Among the potential candidates to interact with CDH13 in *trans* is the GABA-Aα1 subunit, expressed in PV-positive hippocampal interneurons and whose association with CDH13 has already been described in vascular endothelial cells.^67^ Transmembrane molecules of the presynaptic (or postsynaptic) compartments that could potentially interact with CDH13 include integrin-linked kinase,^68^ integrins^67^ or receptor tyrosine kinases,^4^ among others. Such interactions may induce downstream changes in actin cytoskeleton organization,^69^ which destabilize the GABAergic synapse.

*Cdh13* knockdown in dissociated primary hippocampal neurons was previously reported to lead to a reduced density of both excitatory and inhibitory synapses, as well as a decrease in miniature excitatory postsynaptic currents amplitude and frequency.^30^ In the present study, we show in organotypic slices that knockdown using the same shRNA exclusively in excitatory CA1 pyramidal cells did not affect inhibitory or excitatory input onto these cells, consistent with the expression pattern of CDH13 restricted to hippocampal CA inhibitory but not excitatory neurons. In addition, we show that in *Cdh13*^−/−^ mice, miniature excitatory postsynaptic currents amplitude and frequency are unaffected. Together, our data confirm that excitatory synapses are not directly (via shRNA-mediated knockdown of *Cdh13*) or indirectly (secondary to changes in interneuronal activity and/or connectivity) affected. The difference between the two studies may thus be caused by the type of experimental model (postnatal brain sections and organotypic slices vs dissociated cells) rendering it difficult to accurately extrapolate conclusions from both studies.

The hippocampus is central to cognition, spatial navigation, as well as memory formation and specifically the regulation of emotional memories.^35^ Furthermore, altered verbal and visuospatial working memory is one of the neurocognitive alterations widely described in ADHD.^70, 71, 72^ Other mouse models for neurodevelopmental disorders, such as Down syndrome or neurofibromatosis type 1, also present increased inhibition, which correlates with spatial memory deficits.^42, 73, 74, 75^ Remarkably, the excitation/inhibition imbalance triggered by the loss of CDH13 function in the hippocampus was consistently reflected in the behavior of CDH13-deficient mice. We showed that *Cdh13* null mutants display alterations in cognition and learning/memory (particularly, spatial memory deficits during the reversal phase of the Barnes maze when the position of the escape box is changed), suggesting either reduced cognitive flexibility in *Cdh13*^−/−^ mice (that is, inability to change their search strategies during the reversal phase) or a learning deficit when the difficulty of the task increases. In addition, *Cdh13*^−/−^ mice display other minor alterations in conditioned fear responses as well as in the initial phase of the 5-CSRTT, which support the notion of learning deficits as a consequence of CDH13 deficiency. For example, the increased number of timeout responses during the training session may also support the notion of cognitive inflexibility in these mice.^76^ By contrast, during the 5-CSRTT, no modifications in attention or impulsivity were detected in *Cdh13*^−/−^ mice. In the context of neurodevelopmental/psychiatric disorders, this suggests that the contribution of *CDH13* variation to these disorders would be restricted to certain behavioral dimensions, that is, learning ability and cognitive flexibility, whereas other aspects (for example, attentional processes) would be less affected by *CDH13* variation, at least under baseline conditions.

The alteration in cognitive functions observed in *Cdh13*-deficient mice are in general mild but not unexpected, given the observation that single genetic risk variants for neuropsychiatric disorders tend to display very modest effect sizes.^77^ Moreover, considering that deficits and impairments associated with neurodevelopmental disorders, such as ADHD, are more poorly compensated during childhood, the effects of CDH13 deficiency on mouse behavior might even be more pronounced in adolescent mice compared with adult animals investigated in our study. Finally, it is also possible that CDH13 deficiency may induce more extensive inhibitory dysfunction under conditions of environmental adversity, similar to its role as a cardioprotector under acute or chronic cardiac stress or its function in angiogenesis and tumor growth in a mouse model of mammary tumor.^38, 78^ Further studies will be necessary to disentangle the contribution of CDH13 under different environmental conditions in more detail. Finally, we provided evidence that *Cdh13*^−/−^ mice display increased locomotor activity, one of the core phenotypes in ADHD,^79^ which is even more pronounced in mice following adaptation to the environment, suggesting that CDH13 deficiency may impair the hippocampal-dependent learning process that underlies the habituation to consecutive behavioral tests.^80^

We are aware that our study presents some limitations. Although the current work focused on CDH13 in the hippocampus, we and others have previously reported that CDH13 is also expressed in other regions of the mouse brain.^4, 29^ Therefore, the use of conditional *Cdh13*-deficient mice will clarify whether inactivation of CDH13 in other brain regions and neuronal cells impacts at the synaptic and behavioral levels. A cell-specific, such as PV- or SOM-specific, *Cdh13* knockout will facilitate dissection of the specific contribution of CDH13 to each interneuron type where it is expressed. Furthermore, following this line of reasoning, *Cdh13* is also expressed in serotonin (5-HT)-specific neurons of the raphe complex.^4^ Genetic variants of *CDH13* have been associated with criminal behavior with extreme violence,^23^ a domain that is profoundly influenced by the brain 5-HT system.^81^ However, we did not find any indication for alterations in phenotypes related to emotion regulation in *Cdh13*-deficient mice including baseline levels of the stress hormone corticosterone. This also excludes the possibility that observed alterations in the cognitive domain are masked by emotional dysregulation. Finally, no functional *CDH13* variants associated with violent behavior have been identified yet and it is still unknown whether the nature of these variants results in a loss or a gain of CDH13 function.

In conclusion, our findings demonstrate that CDH13 localizes to the presynaptic compartment of hippocampal GABAergic synapses and safeguards the excitatory–inhibitory balance in the CA1 region. CDH13 is thus essential for cognitive flexibility function and correct memory formation. CDH13 deficiency results in behavioral alterations associated with symptoms observed in neurodevelopmental disorders such as ADHD, including learning and memory deficits as well as locomotor hyperactivity. The present findings therefore contribute to the understanding of how dysregulation of CDH13 function may predispose individuals to ADHD and related comorbidities.
