# Supplementary material for: Cadherin-13, a risk gene for ADHD and comorbid disorders, impacts GABAergic function in hippocampus and cognition
Source: Transl Psychiatry. 2015 Oct 13;5(10):e655–. doi: 10.1038/tp.2015.152 (PMC4930129; doi:10.1038/tp.2015.152)
Supplement: Supplementary Information [file tp2015152x1.pdf]

Supplementary Information

for

Cadherin-13, a risk gene for ADHD and comorbid disorders, impacts GABAergic function in hippocampus and cognition

Olga Rivero[1]*, Martijn Selten[2]*, Sarah Sich[1]*, Sandy Popp[1], Lucas Bacmeister[1], Elena Amendola[3], Moritz Negwer[2], Dirk Schubert[2], Florian Proft[1], Dominik Kiser[1], Angelika Schmitt[4], Cornelius Gross[3], Sharon M. Kolk[5], Tatyana Strekalova[6], Daniel van den Hove[6], Thérèse J. Resink[7], Nael Nadif Kasri[2]**, Klaus-Peter Lesch[1,6]**.

File content:

Supplementary Materials and Methods …………………………………….….. *pp* 2-18

Supplementary Tables ……………………………………………………………*pp.* 19-20

Supplementary Figures ………..……………………………………………….. *pp.* 21-30

Supplementary References ……………………………………………………*pp.* 31-32

SUPPLEMENTARY MATERIALS AND METHODS

Generation of a constitutive *Cdh13* knockout mouse line

A constitutive *Cdh13* knockout mouse (*Cdh13*$^{-/-}$) was generated through site-specific DNA recombination using a conditional *Cdh13* mouse line (*Cdh13*$^{loxP/loxP}$), as well as a constitutive Cre deleter line, (C57BL/6.C-Tg(CMV-Cre)1Cgn[1] both in a C57Bl/6N background (Figure S1).

For the generation of the *Cdh13*$^{loxP/loxP}$ mouse line, two independent positive ES cell JM8.N4 clones (HEPD0516_5_C09 and HEPD0516_5_F10), containing a *Cdh13* modified locus (*Cdh13* knockout first allele), were purchased from EUCOMM CONSORTIUM ([2]; http://www.eucomm.org). Briefly, this knockout first allele contained a 4.78 kb 5' homology arm, a FRT-flanked cassette driven by *Cdh13* promoter containing both a *LacZ* reporter gene and a neomycin resistance gene for positive selection (both separated by a loxP sequence), 2 loxP sites flanking exon 3 of *Cdh13* gene as well as a 5.88 kb 3' homology arm. To confirm the presence of the second loxP site flanking the exon 3 in these ES clones, long-range PCR was used to amplify the 3' modified homology arm. The presence of the loxP site generates a new restriction site for the restriction endonuclease *Sac*I. Thus, digestion with this enzyme and sequence analysis allowed us to confirm the presence of this modification and therefore the presence of the *Cdh13* modified locus.

Two positive HEPD0516_5_C09 and HEPD0516_5_F10 JM8.N4 clones containing *Cdh13* knockout first alleles were subsequently injected into C57Bl/6N blastocysts. Only one of the resulting chimeric male mice (six animals from clone HEPD0516_5_F10, one from clone HEPD0516_5_C09) was positive for germ-line transmission, and was therefore used as the founder of the *Cdh13* knockout first mouse line. Removal of the FRT cassette to generate the conditional *Cdh13*$^{loxP/loxP}$ mice was achieved by constitutive FLP-mediated recombination: *Cdh13* knockout first offspring were crossed to C57BL/6J congenic constitutive FLP-deleter mice[3] and further backcrossed to C57BL/6N mice for several generations. Subsequently, *Cdh13*$^{loxP/loxP}$ mice were finally intercrossed with C57BL/6N congenic constitutive Cre-deleter mice[1] and additional rounds of breeding were then performed in order to produce animals that were negative for *Cre* transgene.

Mouse genotyping was performed with genomic DNA samples isolated from tail biopsies that were taken from each animal at weaning. Standard PCR protocols were used to amplify the wildtype, knockout first, floxed and knockout alleles of the *Cdh13* gene, as well as to detect the presence of *Cre* or *Flp*. The specific primers used in each

case are indicated in Supplementary Table S1. The amplification program was the following: 94ºC 1min; 2 cycles of 95ºC 15s, 64ºC 15s and 72ºC 1min; 2 cycles of 95ºC 15s, 61ºC 15s and 72ºC 1min; 20 cycles of 95ºC 15s, 58ºC 15s and 72ºC 1min; 10 cycles of 95ºC 15s, 55ºC 15s and 72ºC 1min; 72ºC 10min.

Animal husbandry

All animals were kept in the Animal Core Facility at the University Hospital of Würzburg on a regular 12h light/12h dark cycle in a temperature (21±1°C) and humidity (50±5%) controlled environment. Food and water were provided *ad libitum*. All animals used for the studies came from matings between *Cdh13*$^{+/-}$ males and *Cdh13*$^{+/-}$ females. The use of heterozygote animals for the breeding allows controlling for any potential effects of genotype on maternal care, and ensures an appropriate distribution of the different genotypes among the litters to be used for experiments. Moreover, animals of the three *Cdh13* genotypes (+/+, +/- and -/-) coming from the different available litters were randomly chosen for the different experiments.

Brain dissection for molecular analysis

For quantitative molecular analysis of CDH13 protein levels, as well as for the analysis of neurotransmitter levels, adult mice (between 12 and 16 weeks of age) were anesthetized with a lethal dose of isoflurane and euthanized by cervical dislocation. Afterwards, brains were dissected, immediately frozen in dry ice-cold isopentane and stored at -80ºC until further use. The isolation of brain areas (including complete hippocampus) was conducted by slicing the brains in thick sections on a pre-cooled plate. Brain regions were then separated, frozen in dry ice and stored at -80ºC until needed.

Western blotting

Hippocampal samples were homogenized by sonication in RIPA (Radio-Immunoprecipitation Assay) buffer (Sigma-Aldrich Chemie Gmbh, Munich, Germany), supplemented with protease and phosphatase inhibitor tablets (Roche Diagnostics Deutschland GmbH, Mannheim, Germany). 15 µg of total protein were loaded on 4-12% Bis-Tris polyacrylamide gels (Life Technologies, Darmstadt, Germany), electrophoresed under reducing, denaturing conditions, electroblotted to a 0.45 µm-pore nitrocellulose membrane (Life Technologies) and probed using a goat anti-Cadherin-13 antibody (1:200, R&D systems, Wiesbaden-Nordenstadt, Germany) and mouse anti- α-tubulin (1:5000, Sigma-Aldrich) or rabbit anti-GAPDH (1:2500, Abcam, Cambridge, United Kingdom) antibodies as loading controls. Finally, membranes were incubated in secondary fluorescent donkey anti-goat 680 and donkey anti-mouse

800CW or donkey anti-rabbit 800CW antibodies (1:10000, Li-Cor Biosciences GmbH, Bad Homburg, Germany). Detection and quantification was conducted using the Odyssey infrared imaging system and Image Studio Lite version 3.1 software (Li-Cor Biosciences). CDH13 protein relative levels were normalized to the internal loading control and the data were expressed as percentages of the average control value.

Analysis of hippocampal neurotransmitter levels

Hippocampal concentrations of different neurotransmitters and their metabolites were also detected via high performance liquid chromatography (HPLC). Total hippocampus samples of *Cdh13*$^{+/+}$, *Cdh13*$^{+/-}$ and *Cdh13*$^{-/-}$ mice were homogenized by sonication in buffer containing H_3PO_4 (150 mM) and DTPA [Bis-(2-aminoethyl)-amine-N,N,N',N'',N''-penta-acetic acid; 500 µM] on ice under an argon atmosphere. 140 µL buffer were used per 10mg of tissue. Homogenates were then centrifuged at 19000 rpm for 20 min at 4°C. Supernatants were used for the analysis of neurotransmitters and metabolites on an Agilent 1100 HPLC system (Agilent Technologies, Waldbronn, Germany). According to previously described methods, electrochemical detection (model 1640; Bio-Rad, Munich, Germany) was used for monoamines and fluorescence detection with precolumn derivatization for amino compounds.[4] The analyzed neurotransmitters and metabolites were gamma-butyric acid (GABA), glutamate, norepinephrine (NE), 3-methoxy-4-hydroxyphenylglycol (MHPG), dopamine, (DA), 3,4-dihydroxy-phenylacetic acid (DOPAC), homovanillic acid (HVA), serotonin (5-HT) and 5-hydroxyindoleacetic acid (5-HIAA).

Histological methods

Nissl staining

Slide-mounted 20 µm thick coronal sections of adult mouse brain were rinsed 2x2 min in ddH_2O and afterwards immersed in filtered 0.1% cresyl-violet solution for 8 min. Subsequently, sections were transferred to 70% EtOH for destaining and the process briefly judged by light microscopy. Sections were then transferred to ascending alcohol concentrations and covered with Vitro-Clud and cover glasses.

In situ hybridization

In situ hybridization (ISH) experiments were carried out as previously described.[5] In summary, a probe against mouse *Cdh13* mRNA (GenBank accession: NM_019707) was generated with the following primers: Forward: 5'-TCCATTGTGGTGTCCCCCA-3'; reverse: 5'-TGGGTTGGTGTGGATCTCGA-3'. A probe against mouse *Gad1* mRNA *(GenBank* accession: NM_008077) was produced via amplification with the following

primers: forward: *5´-ACGTGAAGGGATGATGGATG*-3´ (2417-2436); reverse: 5´-*GCGAAGGCTACTGAAGCAAG*-3´ (2917-2888).

cDNA fragments were cloned into Dual promoter PCR®II according to the manual of the TA Cloning® Kit using One Shot® TOP10F' chemically competent *E.coli* for transformation (Life Technologies). Plasmids were isolated following instructions of Wizard Plus SV Miniprep and PureYield™ Plasmid Midiprep (Promega, Mannheim, Germany). *Cdh13* probes were digoxigenin-labelled, *Gad1* probes were fluorescein-labelled using SP6/T7 RNA polymerase (New England Biolabs, Frankfurt, Germany) and 10x DIG/Fluorescein labeling mix (Roche Diagnostics). For later reproduction of probes from linearized plasmid stocks, the protocol for direct purification of PCR products of the NucleoSpin® Extract II kit (Macherey-Nagel, Düren, Germany) was followed. After *in vitro* transcription, RNA probes were cleaned up using the RNeasy Mini® instructions for RNA cleanup (Qiagen, Hilden, Germany)

Snap-frozen native adult mouse brains were coronally sectioned in 16µm-thick slices with a cryostat microtome (Leica Mikrosysteme Vertrieb GmbH, Wetzlar, Germany) and afterwards stored at -80°C until the beginning of the experiment.

Pre-hybridization and hybridization procedures were carried out as described[5], with alcohol rehydratation being omitted for FISH experiments and hybridization temperatures at 58°C for single *Cdh13* CISH and 60°C for double FISH. Stringent post-hybridization washes were carried out (room temperature if not mentioned otherwise): 2 x 10 min in 2x sodium saline citrate buffer (SSC, Sigma-Aldrich), 50% formamide in 2x SSC at 58°C/60°C for 30 min, 2 x 10 min in 2x SSC, RNase A (40 mg/ml, Roche Diagnostics) in RNase buffer (50 mM NaCl, 10 mM Tris, 1 mM EDTA) at 37°C for 30 min, 3 x 2 min RNase buffer without RNase, 30 min RNase buffer without RNase at 58/60°C, and 5 min in wash buffer (wB; 100 mM Tris, 150 mM NaCl, pH 7.5). In the following steps, wB was used for washing and solutions if not mentioned otherwise.

Detection for single CISH: Sections were incubated with 0,5% Blocking Reagent (Roche Diagnostics) for 60 min, briefly treated with 0,3% Triton X-100 and incubated for 60 min with aP-conjugated anti-DIG Fab fragment (Roche Diagnostics) diluted 1:500 in 0.25% blocking reagent, 0.15% Triton X-10. Subsequently, sections were rinsed 2 x 5 min and 5 min with reaction buffer (rB; 100 mM Tris, 100 mM NaCl, 50 mM $MgCl_2$, pH 7.5) before being incubated 16-20 h in the dark with aP substrate (0.4 M 5-bromo-4-chloro-3-indolyl-phosphate (BCIP) and 0.4 M 4-nitro blue tetrazolium chloride (NBT) in rB). Development of the reaction and intensity of the signal were observed

with a light microscope and the reaction stopped by rinsing and mounted with Aquatex (Merck Millipore, Darmstadt, Germany).

Detection for double FISH: Based on previous literature[6], the following protocol was used for fluorescent detection. Sections were treated with 0.5% PE Blocking Reagent (PE block) (Perkin Elmer, Rodgau, Germany) for 60 min and subsequently incubated with POD conjugated anti-Fluorescein Fab fragments (Roche Diagnostics) diluted 1:3000 in 0.5% PE block for 2 h. Subsequently, sections were rinsed 3 x 6 min with TN buffer (0.05% Tween 20 in wB), incubated for 10 min with DNP reagent (from TSA™ PLUS DNP kit, Perkin Elmer) that had been diluted 1:100 in 50% 1x Diluent/ 50% H_2O (Merck Millipore) and rinsed again 3 x 5 min with TN buffer. To detect digoxigenin-labeled probes, slides were subjected to overnight incubation (4°C) with aP-conjugated anti-DIG Fab fragments (Roche Diagnostics) 1:2000 and anti-DNP Alexa Fluor 488 (Life Technologies) 1:500 in PE block. After rinsing 3 x 7 min in TN buffer and 1 x 4 min in TS8 buffer (0.1 M Tris-HCl, 0.1 M NaCl, 0.01M $MgCl_2$, pH 8.0), a reaction with 2-hydroxy-3-naphtoic acid-2'-phenylalanide phosphate and 4-chloro-2-methylbenzenediazonium hemi-zinc chloride salt (HNPP/FR, Roche, 1:100 in TS8 buffer and sterile-filtered before use to remove precipitates) was carried out under coverslips. After 45 min, the outcome was microscopically observed and the HNPP/FR replaced if further development was desired (repeated up to three times). The reaction was stopped by washing 5 min in ddH_2O, and 300 nM DAPI was applied as a nuclear counterstain. Finally, the slides were rinsed, mounted with Fluoro-Gel (Electron Microscopy Sciences, Hatfield, PA, USA) and stored at -20°C.

Immunofluorescence

Adult mice (aged between 12 and 16 weeks of age) were anaesthetized with isoflurane and transcardially perfused with 4% PFA (8 min, pH 6,5) followed by 4% PFA including 0.02% glutaraldehyde (12 min, pH 11). Brains were postfixed in 4% PFA overnight and subsequently incubated in 10% and 20% sucrose solutions before freezing them in dry ice-cooled isopentane. For double immunofluorescence, 40µm free-floating coronal sections were generated in 8 series from 3 brains. Afterwards, one series of each brain was stained with goat CDH13 antibody (1:500, R&D Systems; #AF3264) in combination with one of the following antibodies against different interneuron markers: rat anti-somatostatin (SOM) (1:400, Merck Millipore; #MAB354), rabbit anti-parvalbumin (PV) (1:250, Swant, Marly, Switzerland; #PV25), rabbit anti-calbindin (CB) (1:800, Swant; #CB-38a), rabbit anti-neuropeptide Y (NPY) (1:100, Sigma-Aldrich; #N9528), rabbit anti-vasoactive intestinal peptide (VIP) (1:300, Immunostar, Hudson, WI, USA; # 20077), rabbit anti-neural nitric oxide synthase (nNOS) (1:250, Merck

Millipore; AB5380), rabbit anti-calretinin (CR) (1:4000, Swant; #7699/3). Subsequently, sections were incubated with secondary fluorescent antibodies (Life Technologies): donkey anti goat-Alexa Fluor 555 (1:600), donkey anti-rabbit-Alexa Fluor 488 (1:1000) and donkey anti-rat-Alexa Fluor 488 (1:800). 1x PBS was used for all washing steps and as diluent for all blocking (10% normal horse serum, 0.25% Triton-X 100) and antibody solutions (5% normal horse serum, 0.125% Triton-X 100). Primary antibody incubation was carried out overnight at 4°C, secondary antibodies were incubated 2 h at room temperature. Slides were mounted with Fluoro-Gel and coverslipped to be stored at 4°C. The same staining principle and antibodies were applied for triple staining of CDH13 with PV and SOM. In this case secondary antibodies were: donkey anti goat-Alexa Fluor 555 (1:600), donkey anti-rabbit-Alexa Fluor 350 (1:1000) and donkey anti-rat-Alexa Fluor 488 (1:800).

Immunohistochemistry

For single immunohistochemical staining of interneuronal GABAergic markers, native brains of adult mice (12-16 weeks of age, *n*=7-9 per genotype) were extracted after euthanizing the animals with an overdose of isoflurane. Brains were postfixed in 4% PFA over 48 h, and cryoprotected and frozen as described above. The brains were sectioned in 50 µm free-floating coronal sections in 6 series covering the whole dorsoventral axis of the hippocampus. Free-floating immunohistochemistry was carried out as previously described.[7] Heat induced epitope retrieval was performed for 20 min and blocking with 10% normal goat serum, 0.25% Triton-X 100 for 90 min. One complete series of free-floating sections was stained with one of the following primary antibodies: rat anti-SOM (1:1000, Millipore), rabbit anti-PV (1:12000, Swant) and rabbit anti nNOS (1:6000, Millipore) in 5% normal goat serum, 0.25% Triton X-100. Biotinylated secondary anti-rabbit or anti-rat antibodies (1:1000, Vector Laboratories, Burlingame, CA) were incubated for 2 h, followed by incubation in AB complex (each 1:100, 90 min, Vector Laboratories) and 3,3'-Diaminobenzidine (DAB) (Roche Diagnostics) until the staining developed to the desired intensity. Brains were excluded for subsequent analysis when sections were damaged severely, so that the investigation was not reliable, or when the staining quality was not sufficient for proper assessment.

For single CDH13 staining, adult mice (12-16 weeks of age) were anaesthetized with isoflurane and transcardially perfused with 4% PFA (8 min, pH 6.5) followed by 4% PFA including 0.02% glutaraldehyde (12 min, pH 11.0). Brains were postfixed in 4% PFA overnight and subsequently cryoprotected and frozen as described above. Brains were coronally sectioned into 20 µm-thick slices with a cryostat microtome and thaw-

mounted onto slides. Sections were treated as indicated in the instructions for N-Histofine Simple Stain Mouse MAX PO (G) (Nichirei Biosciences, Tokyo, Japan). Briefly, sections were blocked with 10% normal rabbit serum, CDH13 primary antibody was diluted 1:500 in 1xPBS and incubated at 4°C overnight. 1x PBS was used for all washing steps and other solutions. DAB was used as a substrate for peroxidase coupled to the secondary antibody polymer supplied in the Histofine kit.

In this case, the use of sections from *Cdh13*$^{-/-}$ animals as negative controls allowed us to better adjust the conditions for the immunohistochemical staining and therefore to ensure that the observed signal is completely specific and originates from CDH13 protein (Figure S1).

Immunocytochemistry

Primary hippocampal neuron cultures were fixed using 4% paraformaldehyde/4% sucrose in PBS and stored at 4°C in PBS. Cells were permeabilized, blocked for 1h and incubated with primary antibody overnight. Secondary antibody was applied for 1h and cells were mounted using DAKO fluorescent mounting medium. Primary antibodies against the following proteins were used: CDH13 (1:500, Millipore, Darmstadt, Germany, ABT121); vesicular glutamate transporter (VGLUT) (1:100, Synaptic Systems, Göttingen, Germany, 135311); vesicular GABA transporter (VGAT) (1:200, Synaptic Systems, 131011); postsynaptic density protein 95 (PSD95) (1:50, Thermo Scientific, Waltham, MA, USA, MA1-045); gephyrin (1:500, Synaptic Systems, 147111); GABA-Aα1 (1:250, Synaptic Systems, 224211). Secondary antibodies were goat anti-rabbit-488 or goat anti-mouse-568 (1:2000, Invitrogen).

Picture processing

Light microscopic images were obtained with an Olympus BX51 microscope and CX9000 camera (Olympus, Hamburg, Germany) using Neurolucida software v.11 (MBF Bioscience, Willsiton, VT, USA). Pictures were taken with 10x or 20x objectives and processed for overall brightness and contrast with ImageJ v. 1.47 or Photoshop 9.0 (Adobe Systems Software Ireland Limited, Dublin, Ireland).

Fluorescence images were obtained using an inverted epifluorescence Olympus IX81 microscope with a XM10 camera (Olympus) and cellSens Dimension 1.4 software (Olympus). Pictures were taken with 10x and 20x objectives and processed with ImageJ or Photoshop. Figures containing images were composed using Photoshop and Illustrator 12.0 (Adobe).

Confocal images were acquired using a Fluoview FV1000 confocal microscope (Olympus). Pictures were taken with a 63x objective and processed for overall brightness and contrast with Photoshop 12.1 (Adobe).

Colocalization assessment

Of each series, only 7-8 sections contained the stratum oriens (SO) and were selected for coexpression assessment. Single-channel fluorescence images, taken from each section to cover the complete dorsoventral extent of the hippocampal SO (corresponding to DAPI, CDH13 and one of the GABAergic interneuron markers), were subsequently merged and combined in a stack. The ImageJ "Cell Counter" plugin was used to identify and count the number of GABAergic positive cells as well as the CDH13 positive cells in each single channel image separately. Finally, those cells that were coexpressing both proteins were identified by showing all marked cells and also counted. It was verified in the single channel, as well as the merged images that the marked cells indeed coexpressed both proteins. Coexpression percentages of each marker with CDH13 were calculated. It should be noted that coexpression of both markers in one cell was only considered if the cell somata could be clearly identified in both the red and the green channels. Despite this method can underestimate the actual degree of coexpression, this conservative approach also reduces the possibility of false positives and can be therefore considered to show the lower level of the coexpression range.

After the countings, sections were assigned to ventral or dorsal hippocampus, considering a section to be the beginning of the ventral hippocampus when the corpus callosum was separated and no longer connecting both hemispheres. All sections before that point were considered as dorsal hippocampus (interaural 1.26-1.34 mm, according to Paxinos and Franklin[8]).

Quantification of immunolabeled cells

DAB-stained sections from 7-9 different brains per genotype (PV: *n= 8* $Cdh13^{+/+}$, *7* $Cdh13^{+/-}$, *7* $Cdh13^{-/-}$; *SOM/nNOS: n=9* $Cdh13^{+/+}$, *8* $Cdh13^{+/-}$, *7* $Cdh13^{-/-}$) were analyzed using Stereo Investigator imaging software v.11 (MBF), which allows the application of a stereological approach to evaluate the number and density of PV-, SOM- and nNOS-positive GABAergic cells in each brain. Particularly, 8-9 sections corresponding to one complete series of a total of six, covering the whole dorsoventral axis of the hippocampal SO, were counted per mouse. The region of interest, in this case the SO,

was traced at 4x magnification in each section, whereas the cells where counted at 10x (nNOS) or 20x (PV, SOM) magnification, using the optical fractionator method. In coherence with the coexpression study, a differentiation between dorsal and ventral hippocampus was made. The volume (mm^3) of the SO, the estimated population numbers of PV-, SOM-, and nNOS-positive cells were calculated for each brain using the section thickness, number of sections and section evaluation interval. The numerical density (cells/mm^3) was calculated form the volume and population number given in the output data from Stereo Investigator. The experimenter was blind to the genotype of the animals at all times of the experiment. Statistics were calculated with Prism 6.04 (GraphPad Software, La Jolla, CA) using Kruskal-Wallis test.

RNA isolation and semiquantitative RT-PCR (qPCR)

Total RNA from brains of different ages was extracted with TRIreagent (Sigma-Aldrich) and 1-2µg total RNA was used for cDNA synthesis using the RevertAid H-minus first strand cDNA synthesis kit (Thermo Scientific). A 1:15 dilution of cDNA pool was used in a 10µl reaction for qPCR analysis using the Sensifast SYBR no ROX qPCR kit (Bioline, Luckenwalde, Germany) and a Rotor-GeneTM 6000 real-time analyzer (Qiagen). qPCR program used was (2 min 95^oC , (5 s 95^oC, 10 s 60 or 65^oC and 15 s 72^oC)x40 cycles). The intron-spanning primers for *Cdh13* used are: Forward GCTTGCTGCTGCTCTTCTC, reverse CTTGGGAGTCAAGCTTCAGG. As reference transcripts, peptidyl prolyl isomerase (*Ppia*) (Forward AGCCATGGAGCGTTTTGGGTCC, reverse AGCAGATGGGGTAGGGACGCT) and β-actin (*Actb*) Forward CTGACCCTGAAGTACCCCATT, reverse AGAGGCATACAGGGACAGCA) were used. qPCR data were analyzed by using comparative quantitation and the relative Q-values of *Cdh13* calculated by equalizing the lowest Ct value to 1. The normalization factor for the reference genes was determined using the GeNORM program (medgen.ugent.be/genorm) and used to normalize the Q-values. Individual experiments were performed in triplicate with three independent mouse brain RNA pools.

Electrophysiology

Mice (P20-22) of both genders were anesthetized with isoflurane before decapitation. 350 µm-thick horizontal slices of the ventral hippocampus were prepared (HM650V vibratome, Thermo Scientific, Waltham, MA USA) in ice cold ACSF containing (in mM): 87 NaCl, 11 Glucose, 75 Sucrose, 2.5 KCl, 1.25 NaH_2PO_4, 0.5 $CaCl_2$, 7 $MgCl_2$, 26 $NaHCO_3$, continuously oxygenated with 95% O_2/5% CO_2 and incubated for 1 h at 32°C after which they were allowed to cool down to room temperature. Before recording,

slices were transferred to the recording setup and incubated in recording artificial cerebrospinal fluid (ACSF) containing (unless otherwise stated) (in mM): 124 NaCl, 1.25 NaH_2PO_4, 3 KCl, 26 $NaHCO_3$, 10 Glucose, 2 $CaCl_2$, 1 $MgCl_2$ and continuously oxygenated with 95% O_2/5% CO_2 at 30°C and incubated 15 minutes prior to recording. Cells were visualized with an upright microscope (Olympus). Patch pipettes (3-5 MΩ) were made from borosilicate glass capillaries and filled with intracellular solution containing (in mM): 115 $CsMeSO_3$, 20 CsCl, 10 HEPES, 2.5 $MgCl_2$, 4 Na_2ATP, 0.4 NaGTP, 10 Na-Phosphocreatine, 0.6 EGTA (pH 7.2-7.3, 285-295 mOsm). Traces were recorded using a Multiclamp 700B amplifier (Molecular Devices, Wokingham, United Kingdom), sampled at 10 kHz and filtered at 2 kHz. Cells were excluded from analysis if the access resistance exceeded 25 MΩ. Miniature inhibitory postsynaptic currents (mIPSCs) were recorded in the presence of Tetrodotoxin (1 µM), 6-Cyano-7-nitroquinoxaline-2,3-dione (CNQX, 5 µM) and D-(-)-2-Amino-5-phosphonopentanoic acid (D-APV 100 µM). Miniature excitatory postsynaptic currents (mEPSCs) were recorded in the presence of TTX and Picrotoxin (PTX, 100 µM). Paired pulse ratio (PPR) was recorded in the presence of CNQX and D-APV, the recording ACSF contained 4 mM $CaCl_2$ and 4 mM $MgCl_2$, and was calculated as peak2/peak1 after correcting for any residual current at the second pulse. Miniature recordings were analyzed using Mini Analysis Program (Synaptosoft, Decatur, GA, USA). Other traces were analyzed using Clampfit 10.2. All drugs were purchased from Tocris (Abingdon, United Kingdom). The experimenter was blind to the genotype of the animals at all times of the experiment.

Intrinsic cell parameters we recorded in current clamp with intrinsic solution containing (in mM): 13 KCl, 117 K-Gluconate, 10 K-HEPES, 1 $CaCl_2$, 2 $MgCl_2$, 11 EGTA, 2 Na_2ATP, 0.5 Na*3*GTP (pH 7.2-7.3, 300-310 mOsm). Traces were amplified with a SEC-05X amplifier (npi, Tamm, Germany), sampled at 10 kHz and filtered at 2 kHz using an CED 1401 digitizer (Cambridge Electronics Devices, Cambridge, UK), and recorded using Signal (CED, Cambridge, England). Traces were analyzed using Stimfit v0.13[9], following the parameter definitions used by Tricoire et al. (2011).[10]

Morphological reconstructions

For morphological reconstructions, the internal solution was supplemented with 0.4% biocytin (Sigma-Aldrich). The brain slices with biocytin-filled neurons were fixated at 4°C in 4% paraformaldehyde in 0.1 M PBS for several days, and subsequently processed following a modified staining protocol based on Marx et al. (2012).[11] Briefly, after fixation, slices were rinsed in 0.1 M PB, incubated in 3% H_2O_2 in 0.1 M PB for 30 min, rinsed in 0.1 M PBS, then incubated in Avidin-Biotin-Peroxidase solution

(Vectastain Elite, with 1% v/v Triton-100) overnight on a shaking platform at 4°C. The next day, slices were washed with 0.1 M PBS and pre-incubated with DAB solution with nickel enhancer (Vector Peroxidase substrate kit, SK-4100) for 30 min. Then, the DAB solution was replaced with the same solution plus H_2O_2, and incubated for ca. 30 sec. Slices were then rinsed in 0.1 M PB, mounted on gelatinized coverslips, and dried for 3-6 h in a custom-made moist chamber at room temperature. The slides were then dehydrated in an ethanol series and xylene, and then sealed with Eukitt (Sigma). Slices were imaged on a Zeiss Axioskop 1 upright brightfield microscope with 20x and 40x objectives (Zeiss EC Plan-Neofluar, NA 0.5 and 0.75, respectively) and motorized stage (MBF). The camera and motorized stage were connected to a desktop computer running Neurolucida v10 (MBF). Cells were selected based on position (in CA1 stratum pyramidale), pyramidal morphology, staining intensity, and patch depth (>50 µm to safeguard against cutting artefacts). Somata, apical and basal dendrites were reconstructed in Neurolucida as three-dimensional drawings overlaid over the video image. Reconstructions were analyzed in NeuroExplorer (MBF) for intrinsic parameters and Sholl analysis.

Hippocampal organotypic slice preparation and infection

Organotypic hippocampal slice cultures were prepared from postnatal day 6 to 8 rat pups as has been described previously (Stoppini, Buchs, & Muller, 1991) and maintained in a 5 % CO2 humidified 35°C incubator on slice culture medium consisting of MEM, 20 % horse serum, 1 mM L-Glutamine, 1 mM CaCl2, 2 mM MgSO4, 0.25 mg Insulin, 1.25 mM Ascorbic acid, 12.87 mM D-Glucose, 5.25 mM NaHCO3 and 30 mM Hepes (pH 7.3, 325 mOsmol). Slices were infected at day in vitro (DIV) 1 by injection of a lentivirus into the CA1 pyramidal cell layer using a PLI-10 pico-injector (Warner Instruments) and used for electrophysiological experiments DIV 12-13. The lentivirus carried an shRNA directed against *Cdh13* (5’ ACGATCGCTACTTATCAAC 3’) that has been previously described (Paradis et al., 2007).

Behavioral assessment

Animals

Mice were group-housed in Macrolon type III cages in age- and genotype-matched pairs. Prior to the beginning of the behavioral testing, mice were allowed to adapt to the test room for two weeks. The sequence of experiments was designed to minimize the suffering and stress of the animals, beginning with less stressful measurements and finishing with more demanding ones. Behavioral tests were separated from each other

by at least one week. All experiments were performed during the light phase (10:00-16:00. Behavioral test batteries lasted for approximately 8-12 weeks. The experimenter was blind to the genotype of the animals at all times of the experiment.

Beginning at 10 weeks of age, one cohort of mice (7 *Cdh13*$^{+/+}$, 8 *Cdh13*$^{+/-}$, 7 *Cdh13*$^{-/-}$) was tested for anxiety-like behavior in the elevated plus maze (EPM), marble burying test (MBT) and light/dark box (LDB) as well as locomotor activity in the open field (OF) and social interaction (SI) in a test for sociability and preference for social novelty. The OF test was repeated in an independent cohort of age-matched mice (10 *Cdh13*$^{+/+}$, 10 *Cdh13*$^{+/-}$, 10 *Cdh13*$^{-/-}$). This cohort was further subjected to the novelty-suppressed feeding test (NSF) to assess anxiety-like behavior, the Porsolt swim test (PST) to measure behavioral despair, and a delay fear conditioning paradigm to examine associative learning and memory. Another naïve cohort of 15-18 week old mice (7 *Cdh13*$^{+/+}$, 9 *Cdh13*$^{+/-}$, 10 *Cdh13*$^{-/-}$) was tested for visuospatial learning and memory in the Barnes maze (BM) and behavioral despair in the PST. Finally, beginning at 32 weeks of age, a fourth batch of mice (10 *Cdh13*$^{+/+}$, 10 *Cdh13*$^{+/-}$, 10 *Cdh13*$^{-/-}$) was subjected to the 5-choice serial reaction time task (5-CSRTT) to assess attentional performance and impulsivity. In all cohorts, body weights as well as food and water intakes were monitored weekly throughout the entire testing period. In addition, mice of the 1st and 3rd cohort were used to determine baseline stress hormone levels from fecal samples and blood as well as anhedonia-like behavior in a sucrose preference test (SPT).

Sucrose preference

Prior to the beginning of the behavioral test battery, mice were given concurrent access to one bottle of 1% (w/v) sucrose solution and one bottle of tap water for 48 h. The bottles were weighed after 24 h and their positions switched in order to minimize possible effects of side-preference. Sucrose preference was calculated as the percentage of sucrose solution consumed relative to total liquid intake, with a reduced sucrose preference being an indicator of anhedonic behavior.

Elevated plus maze

Mice were individually placed in the center of a maze in the shape of a cross[12, 13], elevated approximately 60 cm from the floor and consisting of two closed, dark arms (15 cm high walls, illumination of 5 lx) and two open arms (illumination of 50 lx), all of them with a dimension of 30x5 cm and semi-permeable to infrared light (TSE Systems, Bad Homburg, Germany).[14] The duration of the test was 10 min, and during this time the animals were tracked with a CCD camera and their behavior was analyzed by

VideoMot2 software (TSE Systems). Particularly, the software was used to calculate the total distance traveled as well as the number of entries and time spent in the open arms as measures of anxiety-like behavior.

Light-dark box

The light-dark box (LDB) consisted of an opaque white box (50x50x40 cm) with a black insert, measuring one third of the total box size and semi-permeable to infrared light (TSE Systems). The illumination in the dark compartment was between 0 and 5 lx, whereas the illumination in the lit compartment was approximately 100 lx. Each mouse was individually placed in the dark section of the box and behavior was automatically recorded with VideoMot2 (TSE Systems) for 10 min. The analyzed measures were distance traveled as well as number of entries and time spent in the lit compartment as measures of anxiety-like behavior.

Marble burying

The marble burying test is a common test to assess anxiety- and compulsive-like behaviors. Mice were individually placed into cages (42.5x26.6x15.5 cm) filled with clean bedding to a depth of 5 cm and 15 identical glass marbles that were evenly spaced across the bedding surface. After 30 min, mice were returned to the home-cage and all marbles covered by bedding to two-thirds or more were scored as buried.

Novelty-suppressed feeding

The novelty-suppressed feeding (NSF) test is based on a conflict between food-seeking induced by hunger and the aversion to explore a novel, brightly illuminated environment.[15] Food-deprived (~20 h) mice were placed in a corner of a brightly lit arena (50x50x40 cm; the same box that was used for the light/dark test) with a single food pellet (~2.5 g) located at the center. After the first feeding event or a maximum duration of 10 min, mice were individually placed in a new home-cage for 5 min and allowed to eat a pre-weighed food pellet. The latency to feed was recorded as a measure of anxiety-like behavior. The amount of food consumed in the home-cage and the body weight loss during the food deprivation period was calculated to determine the animal’s appetitive drive.

Open field

The open field (OF) consisted of an opaque quadratic box (50x50x40 cm) semi-permeable to infrared light (TSE Systems) with an illuminated floor (between 50 and 100 lx from the walls to the center of the arena).[14] Mice were individually placed in one corner of the arena and their movements were automatically recorded during a 30-min period using an infrared-sensitive CCD camera that was positioned above the center of

the box and was controlled by VideoMot2 software (TSE Systems). This software was also used to evaluate the distance traveled and the number of vertical rears as measures of locomotor activity, as well as the time each mouse spent in the central area as a measure of anxiety-like behavior.

Sociability and preference for social novelty

The test apparatus was of a black quadratic open field (50x50x40 cm) semi-permeable to infrared light (TSE Systems). Mice were habituated to the testing chamber during the 30-min OF test, which was performed prior to the 2-trial social interaction test. In the first 10-min trial (sociability test), mice were given a choice between exploring an unfamiliar mouse that was enclosed in a small wire cage or an empty cage. The small cages were located in the upper left and upper right corner of the OF and the position of the cage containing the stranger mouse vs. the empty cage was randomly alternated between animals to prevent possible effects of side preference. In the second 10-min trial (test of preference for social novelty), mice were given a choice between exploring the first, now familiar mouse or a novel, unfamiliar mouse. Behavior was recorded and analyzed with VideoMot2 (TSE Systems). Measures taken included the total distance traveled as well as the number of visits and time spent in each interaction zone (empty vs. mouse and familiar vs. novel, respectively).

Barnes maze

The Barnes maze was performed to assess visuospatial learning and memory. The apparatus consisted of a circular grey platform (120 cm in diameter) with 40 holes around its circumference (each with 5 cm in diameter) with one of them containing an escape box (TSE Systems). Visual cues were located around the platform to allow visuospatial orientation. Each mouse was individually placed in the center of the maze and behavior was recorded until the mouse found the escape box or for a maximum duration of 3 min. Mice were trained to find the hidden box for 8 days (18 trials; acquisition phase). Afterwards, the position of the escape box was changed and the mice were tested for 5 additional days (12 trials; reversal phase). Behavior was automatically recorded using VideoMot2 (TSE Systems) and various parameters were measured, namely latency to escape, distance traveled and velocity. The number of primary errors, defined as the number of times a mouse poked its head around a wrong hole before the first encounter with the escape hole, were manually scored.

5-choice serial reaction time task

The 5-choice serial reaction time task (5-CSRTT) was used to assess attention and impulsivity.[16] Mice were trained in an operant conditioning chamber (20x15x15 cm),

enclosed within a housing and connected to a computer system with software (OBS V3.07, TSE Systems). The rear wall of the chamber had a curved shape and carried an array of five stimulus holes (2 cm in diameter, 3 cm deep and 1.5 cm above the floor), each equipped with an infrared photocell beam to detect nose-pokes, a LED stimulus light to provide illumination of the hole, and a pellet dispenser to deliver a 20 mg dustless food pellet (Bio-Serv, Flemington, NJ) directly into the hole. In addition, the chamber was equipped with a house light (30 lx) mounted in the front wall.

Food restriction:

Throughout testing, mice were maintained at 80-85% of their free feeding weight by restricting the access to food to 1.5-2.5g/day/mouse (depending on the daily weight loss and the number of pellets eaten during testing). Food restriction started 4 days prior to the beginning of the experiment.

Habituation:

During habituation, mice were familiarized with the testing chamber and trained to associate nose-poking into a lit hole with reward. Each trial started with simultaneous illumination of all five holes and immediate delivery of two food pellets into each hole (10 pellets in total) and lasted for 5 min or until the mouse made a nose-poke in each of the five holes. Mice received 8 habituation sessions on 4 consecutive days.

Autoshaping:

In 5 autoshaping sessions (1 session/day), mice were trained to discriminate between nose-pokes that lead to reward (lit holes) and those that do not (unlit holes). A session was terminated after 30 completed trials or a maximum duration of 20 min. Each trial started with the illumination of one of the five holes (in pseudo-random order). The hole remained illuminated until a response was made into that hole. If an animal made a nose-poke into the lit (correct) hole, a pellet was delivered immediately and the stimulus light was extinguished. The next trial was initiated after an intertrial interval (ITI) of 5 s. Responses into unlit holes were counted but had no further consequences. Parameters measured included the total number of nose-pokes, the average latency to complete a trial (correct response latency), the number of trials completed, the number of pellets eaten, and the total duration of the session.

Training:

During acquisition of the 5-choice visual discrimination task, mice were trained for one session/day. Each session started after a 5 s habituation period and terminated after 60 trials or a maximum duration of 20 min. Each trial started with an ITI of 10 s, followed by the illumination of one of the five holes (in pseudorandom order) for a

predefined stimulus duration (SD). The SD was initially set to 20 s and gradually decreased to 10, 5 and 2 s. The SD was followed by a limited hold (LH) period of 2 s, during which the stimulus light was switched off but a nose-poke was still considered a correct response. A nose-poke into the correct hole during the SD or LH was rewarded with a food pellet and followed by the next ITI. A nose-poke into one of the four unlit (incorrect) holes was punished by a timeout period of 5 s, signaled by the illumination of the house light. A response during the last second of the ITI prolonged this interval for another second in order to prevent stimulus presentation while an animal was not able to pay attention.[17] Mice received one training session for the 20 s stimulus and 5 sessions for the 10, 5 and 2 s stimuli. The software automatically recorded the number of correct responses (nose-pokes into lit holes during the SD or LH), incorrect responses (nose-pokes into unlit holes during the SD or LH), premature responses (nose-pokes during the ITI), omission errors (number of trials without response), correct and incorrect response latencies (time between stimulus onset and response), the number of trials completed and the duration of the session. Accuracy was calculated as percentage of correct responses [= correct responses / (correct+incorrect responses) x 100]. Timeout responses were calculated as the difference between the total number of nose-pokes, premature nose-pokes, correct and incorrect nose-pokes.

Porsolt swim test

This test is used to assess learned helplessness as a hallmark of depression-like behavior. Mice were placed in a 1000 ml glass beaker filled with water (~27°C) to a height of 15 cm. Behavior was automatically recorded for 6 min using VideoMot2 (TSE Systems), and the percentage of time each mouse spent floating, swimming and climbing was calculated.

Fear conditioning

An automated fear conditioning chamber (TSE Systems) was used to assess associative fear learning and memory. During conditioning (day 0), mice were subjected to a 60 s habituation phase followed by the presentation of an auditory cue (80 dB, 4 kHz tone; conditioned stimulus, CS) for 20 s that co-terminated with a 0.80 mA scrambled footshock (unconditioned stimulus, US) during the last 2 s. Mice received three CS-US pairings with an intertrial interval of 60 s. On day 1, mice were tested for cued fear memory by presentation of the auditory cue for 180 s in a modified environment. On day 2, mice were exposed to the original conditioning chamber for 300 s to assess contextual fear memory. During the entire experiment, behavioral responses like distance traveled, rearing, activity (the duration of movement above a

speed threshold of 2cm/s), maximum speed, jumping and freezing (complete immobility for a duration of >2s) were automatically recorded via infrared light beams.

Corticosterone measurements

Baseline stress hormone levels were assessed prior to the beginning of the emotionality test battery trough the measurement of corticosterone (CORT) metabolites in fecal samples.[18] For sampling, the mouse cages were changed the day before feces collection and fecal samples were pooled according to genotype (*n*=6-7 samples/genotype consisting of 2 mice/sample). Following collection, fecal samples were stored at -20°C for further use. 0.05 g of the dried sample were extracted using 80% methanol and corticosterone metabolite levels were determined in a 5alpha-pregnane-3beta,11beta,21-triol-20-one enzyme immunoassay.

One week after completion of the behavioral test batteries, mice were anesthetized with isoflurane and blood samples were collected from the heart. Plasma CORT concentrations were determined by enzyme-linked immunosorbent assay (ELISA, Demetitec Diagnostics GmbH, Kiel, Germany) following manufacturer´s instructions.

SUPPLEMENTARY TABLES

Table S1. Primer oligonucleotide sequences used for the genotyping of Cdh13-modified mouse lines

Mouse line /allele	Forward Primer (5'→3')	Reverse Primer (5'→3')	Amplicon size (bp)
Flp	CACTGATATTGTAAGTAGTTTGC	CTAGTGCGAAGTAGTGATCAGG	725
**hCMV-*Cre***	GCGCGGTCTGGCAGTAAAAAC	CGCCGCATAACCAGTGAAACA	117
***Cdh13* ko first**	ATCACGACGCGCTGTATC	ACATCGGGCAAATAATATCG	108
***Cdh13* floxed**	TGGTTCTGCTCCAAGACTCAG	ATTAGGGACTATCCTGGGCTA	437 (fl)<br>233 (wt)
***Cdh13* ko**	TGGTTCTGCTCCAAGACTCAG	CCAGGAAGAGATAAAGCCAGG	478 (ko)<br>1190 (wt)

Abbreviations: bp, base pairs; fl, floxed; ko, knockout; wt, wildtype.

Table S2. Morphological parameters of reconstructed CA1 pyramidal cells in *wt* and *Cdh13*$^{-/-}$ mice

Morphology		*Cdh13*$^{+/+}$	SEM	*Cdh13*$^{-/-}$	SEM	P (T-test)	Significance
**Basal dendrites**	**Quantity**	4.3	0.6	4.6	0.6	0.773	
	**Nodes**	17.0	1.9	13.0	1.4	0.117	
	**Ends**	21.3	2.2	17.6	1.9	0.212	
	**Length(µm)**	1631.4	263.3	1398.2	173.1	0.463	
	**Mean length**	384.9	71.9	314.2	25.5	0.345	
**Apical dendrites**	**Nodes**	31.5	1.6	40.6	1.4	0.001	**
	**Ends**	32.3	1.5	41.9	1.7	0.002	**
	**Length(µm)**	3041.5	436.1	4521.4	478.9	0.046	*
**Convex hull**	**Area (µm²)**	89281	16353	86809	5788	0.959	
	**Perimeter (µm)**	1322	88	1297	37	0.908	

Table S3. Intrinsic properties of CA1 hippocampal neurons

Electrophysiology		*Cdh13*$^{+/+}$	SEM	*Cdh13*$^{-/-}$	SEM	*P* (t-Test)	Significance
Basal properties	V_{Rest}	-61.7	2.0	-60.7	1.3	0.214	
	I_{sag} (mV)	-86.1	2.0	-87.9	2.3	0.082	
	Sag Index	0.8	0.1	0.7	0.1	0.205	
	R_M (MΩ)	166.4	44.7	158.1	42.7	0.689	
	T_c (ms)	21.9	5.3	26.3	4.6	0.065	
Action potentials	APs at Rheobase	6.8	6.9	8.1	5.7	0.639	
	APs at 2x Rheobase	15.6	7.4	16.7	5.5	0.752	
	AP Threshold (mV)	-40.6	2.9	-41.0	4.7	0.830	
	AP Amplitude (mV)	85.5	6.3	90.5	5.0	0.069	
	Rise time (20-80%)	0.2	0.0	0.2	0.0	0.304	
	Half-width (ms)	1.2	0.1	1.2	0.1	0.892	
	Slope rise (mV/ms)	276.2	54.5	306.0	37.1	0.173	
	Slope decline (mV/ms)	66.8	6.6	66.8	7.5	0.989	

SUPPLEMENTARY FIGURES

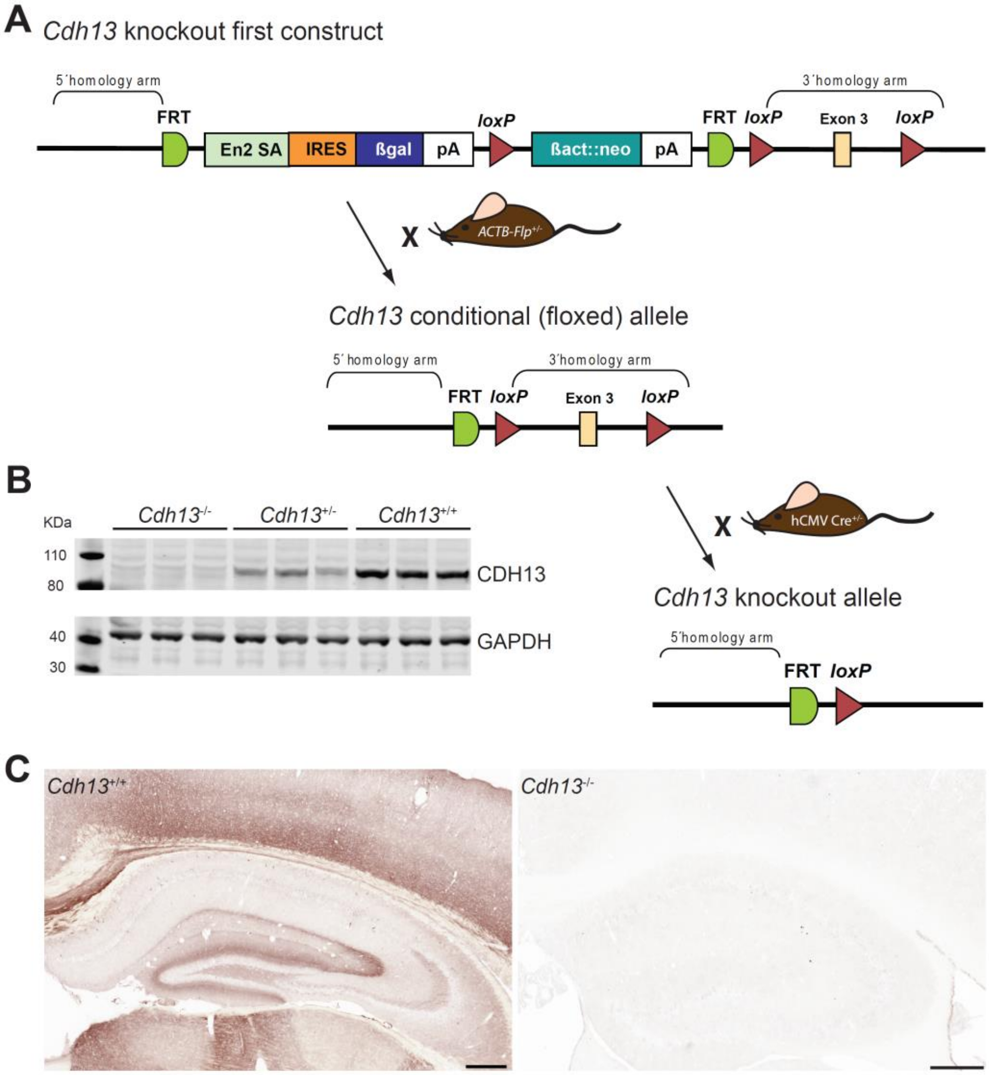

Supplementary Figure S1. Generation of a constitutive *Cdh13* knockout mouse. (A) Schematic representation of the *Cdh13* knockout first targeting construct expressed in ES cells that were injected into C57Bl6/N blastocysts. Afterwards, *Cdh13* knockout first mice were bred with Flp-deleter mice in order to remove the FRT-flanked cassette (containing both the *LacZ* reporter gene and the neomycin resistance gene). The resulting *Cdh13* conditional (floxed) mice were intercrossed with constitutive Cre-deleter mice, which induced the recombination of exon 3 and the generation of a truncated protein in the *Cdh13* knockout (*Cdh13*$^{-/-}$) mice. **(B)** Western blot analysis of mouse brain lysates shows, in wildtype (*Cdh13*$^{+/+}$) mice, a prominent band of approximately 90 kDa corresponding to the mature CDH13 protein. This band could not be detected in *Cdh13*$^{-/-}$ mice, whereas reduced levels were observed in heterozygote (*Cdh13*$^{+/-}$) mice, suggesting a gene dose effect. GAPDH antibody was used to confirm the equal loading in all lanes. **(C)** Absence of CDH13 protein in the brains of *Cdh13*$^{-/-}$ mice was also confirmed by immunohistochemistry. No stained cells or fibers were found in any region of the brain (i.e. cortex or hippocampus). Scale bars 300µm. Abbreviations: FRT, Flippase Recognition Target; En2 SA, splice acceptor of mouse En2 exon; IRES, Internal Ribosome Entry Site; ßgal, ß-galactosidase; pA, polyadenylation sequence; *loxP*, locus of X-over P1; ßact: ß-actin; Neo, Neomicin resistance gene; *ACTB*, ß-actin gene; Flp, Flippase; hCMV, human cytomegalovirus; +, wildtype; -, knockout; GAPDH, Glyceraldehyde 3-phosphate dehydrogenase.

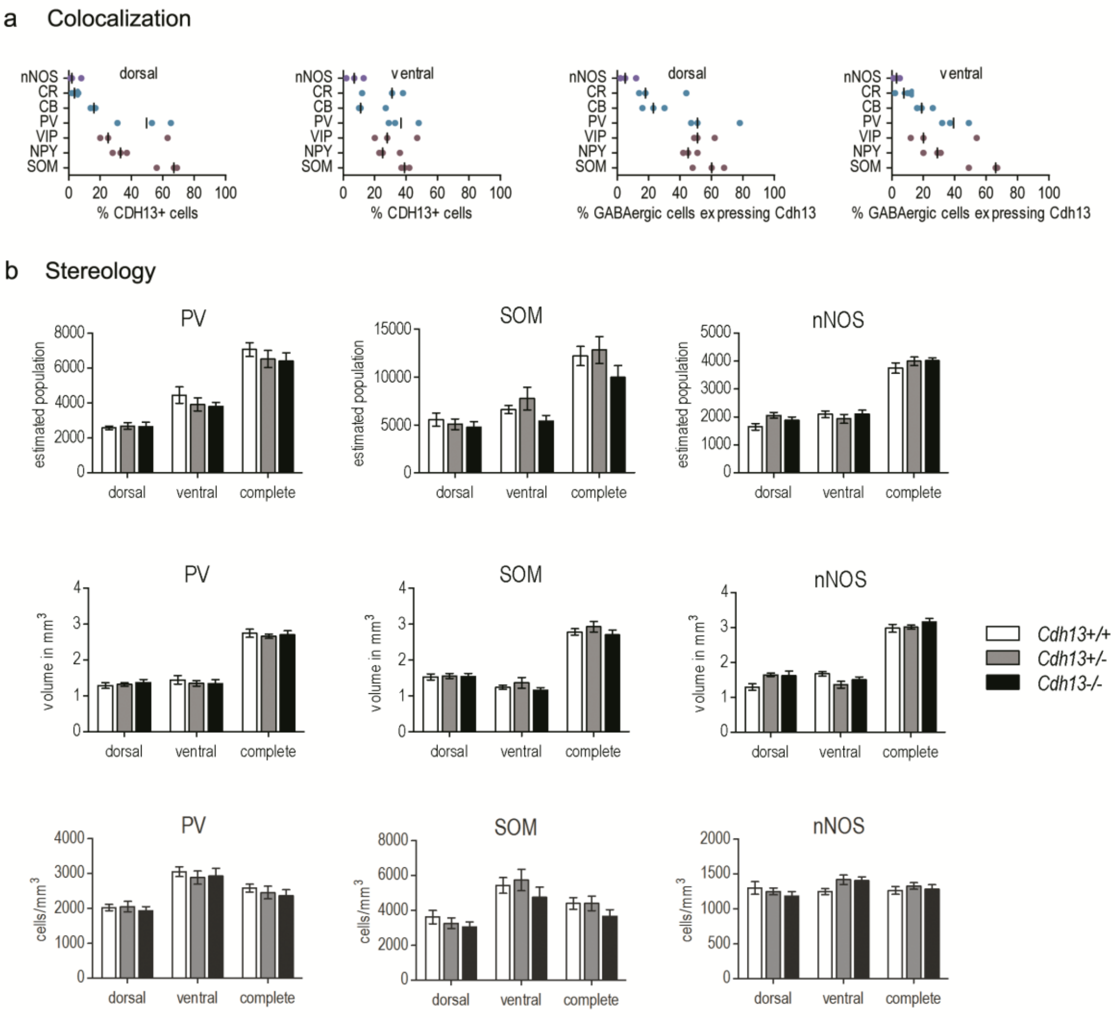

Figure S2. Histological investigation of CDH13 in GABAergic interneurons of adult mouse stratum oriens (SO). **(a)** Colocalization of CDH13 with neurochemical markers of GABAergic interneurons in the SO of the dorsal and ventral hippocampus. Higher colocalization was observed in the dorsal part of the hippocampus, n=3; lines represent median. **(b)** Stereological quantification of PV, SOM and nNOS-positive cells in the SO of *Cdh13*$^{+/+}$, *Cdh13*$^{+/-}$ and *Cdh13*$^{-/-}$ mice; (PV: *n= 8 Cdh13*$^{+/+}$*, 7 Cdh13*$^{+/-}$*, 7 Cdh13*$^{-/-}$*; SOM/nNOS: n=9 Cdh13*$^{+/+}$*, 8 Cdh13+/-, 7 Cdh13)*; bars represent mean of estimated population number (first row), mean SO volume (second row), and mean of cell density (calculated from the population numbers and volumes). Error bars represent SEM; Kruskal-Wallis test revealed no significant differences between genotypes.

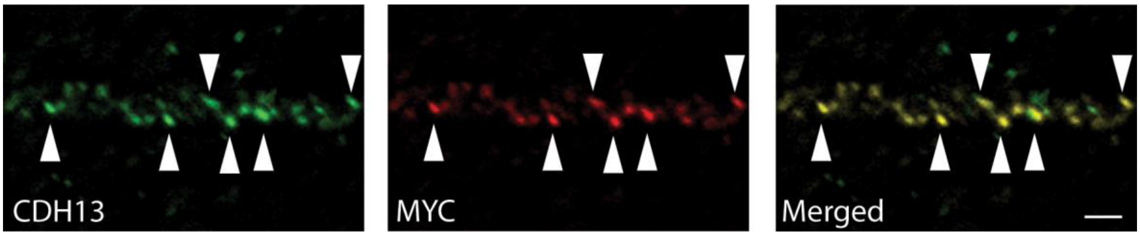

Figure S3. **Confirming the specificity of CDH13 rabbit antibody.** Immunofluorescence staining for CDH13 (green) and MYC (red). Triangles indicate CDH13 and MYC double-positive puncta. Scale bar: 1µm.

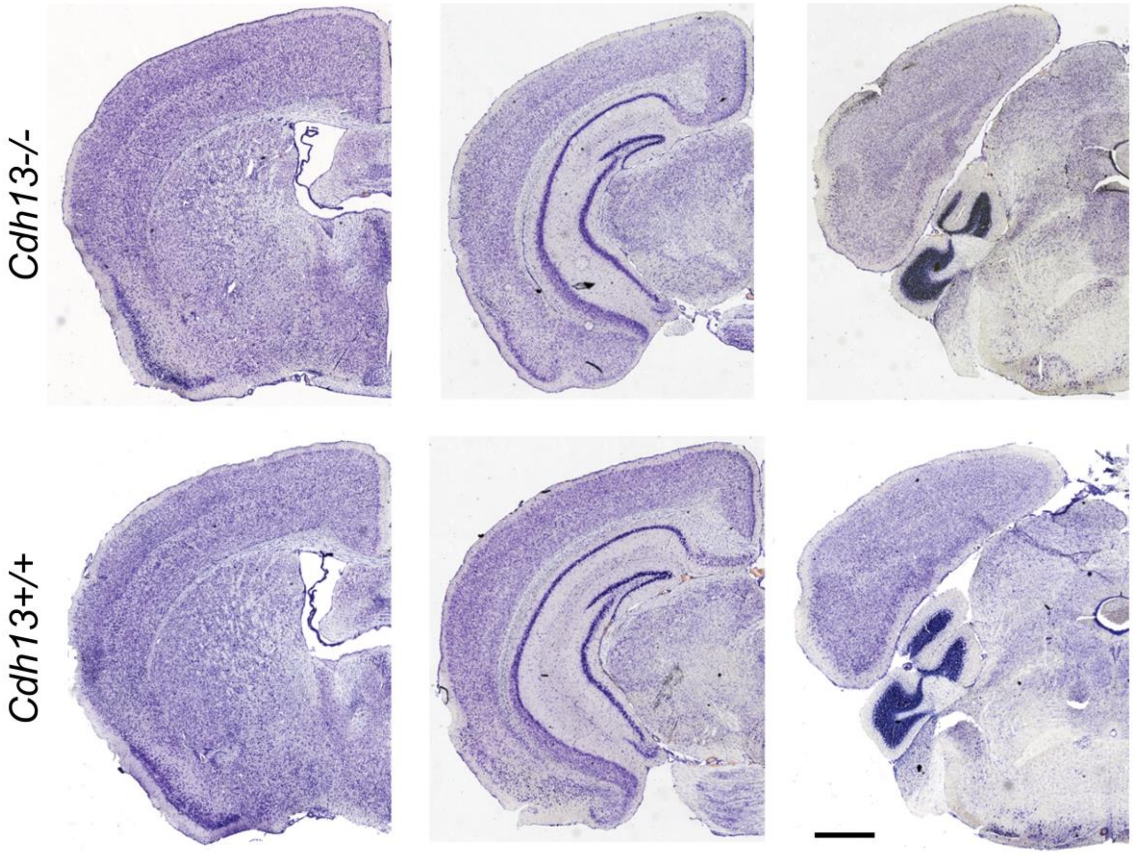

Figure S4. **Nissl staining in *Cdh13*$^{+/+}$ and *Cdh13*$^{-/-}$ adult mouse brains.** No gross morphological defects were detectable in *Cdh13*$^{-/-}$ mice. Scale bar: 500µm.

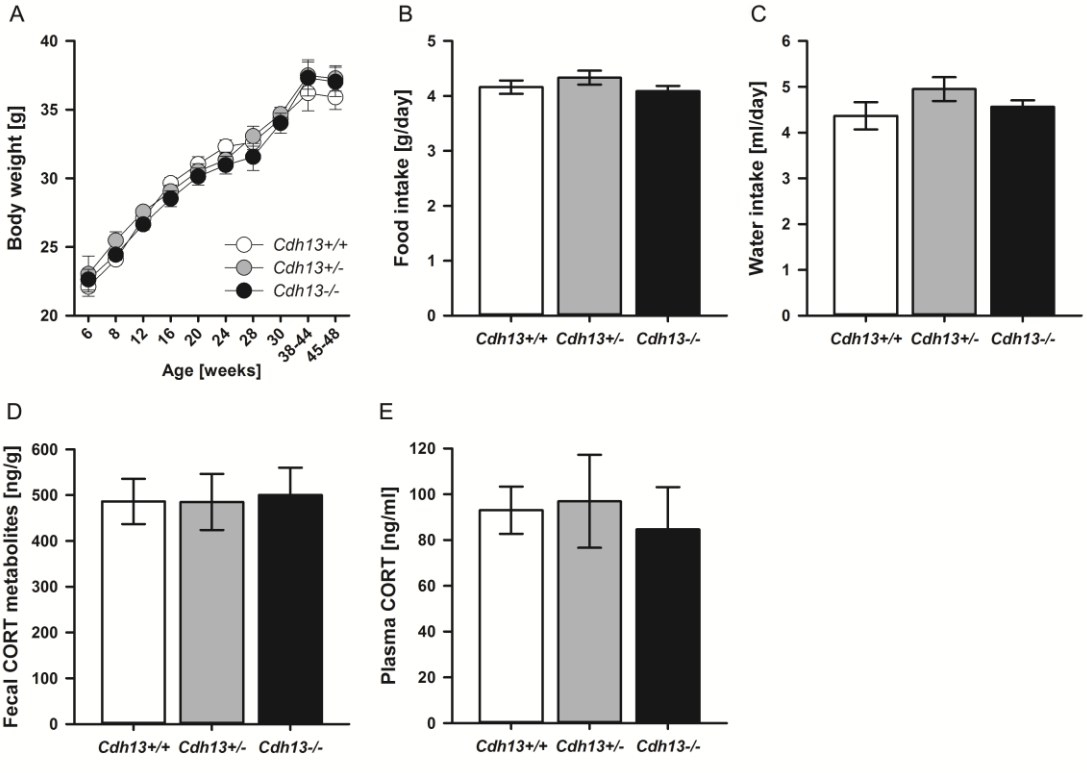

Figure S5. **Body weight gain, feeding behavior and stress hormone levels are not affected by *Cdh13* genotype. (A)** Body weight gain at different stages of age did not significantly differ between *Cdh13*$^{-/-}$, *Cdh13*$^{+/-}$ and *Cdh13*$^{+/+}$ mice (*n*=7-36/genotype). **(B)** Food intake and **(C)** water intake were similar in all three genotypes (*n*=13-15 cages/genotype, 2 mice/cage). **(D)** Fecal CORT (*n*=6-7 cages/genotype, 2 mice/cage) and **(E)** plasma CORT (*n*=6/genotype) levels were not significantly altered in *Cdh13*$^{-/-}$ and *Cdh13*$^{+/-}$ mice as compared to *Cdh13*$^{+/+}$ mice. Data are shown as mean±SEM. Abbreviations: CORT, corticosterone.

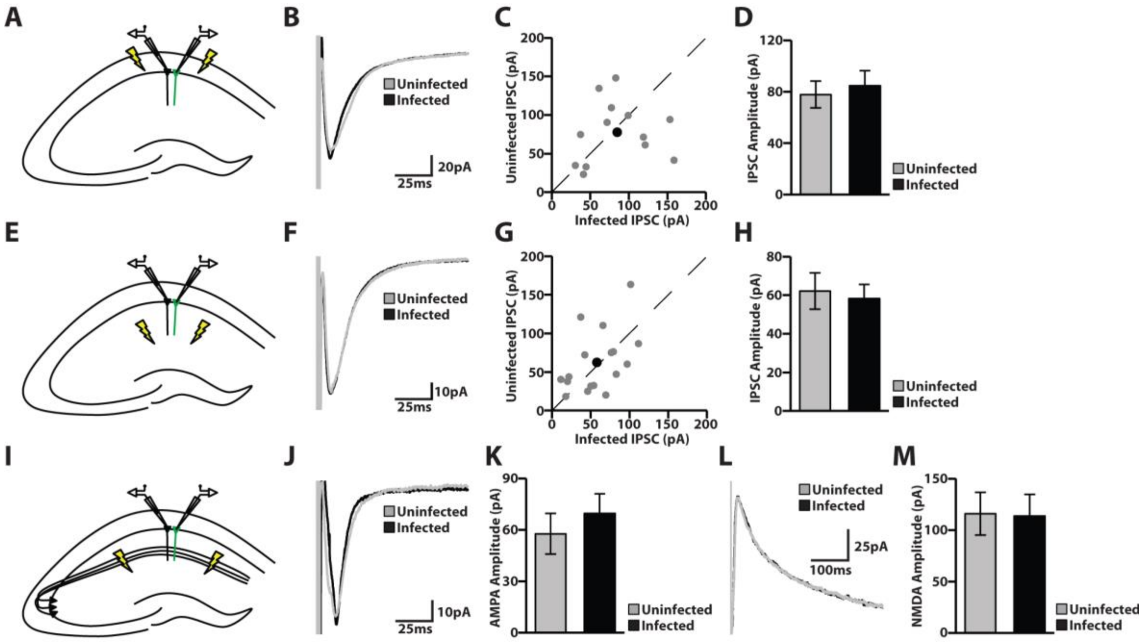

Figure S6. Postsynaptic knockdown of *Cdh13* in CA1 pyramidal cells has no effect on synaptic transmission. (A-D) Stimulation in stratum oriens **(A)** layout experiment **(B)** example trace of IPSC **(C)** IPSC amplitude for each recorded pair (gray dots) and average (black dot) **(D)** bar graph showing average IPSC amplitude (*n*=13/3). **(E-H)** Stimulation in stratum radiatum **(E)** layout experiment **(F)** example trace of IPSC **(G)** IPSC amplitude for each recorded pair (gray dots) and average (black dot) **(H)** bar graph showing average IPSC amplitude (*n*=17/4). **(I-M)** Stimulation of Schaffer collaterals for AMPA- **(J,K)** and NMDA response **(L,M)** **(I)** layout experiment **(J)** example trace of EPSC **(K)** bar graph showing average AMPA amplitude (*n*=13/2) **(L)** example trace of mEPSC **(M)** bar graph showing average NMDA amplitude (*n*=13/2). Bar graphs are shown as mean±SEM. *N* is shown as number of cells/number of animals.

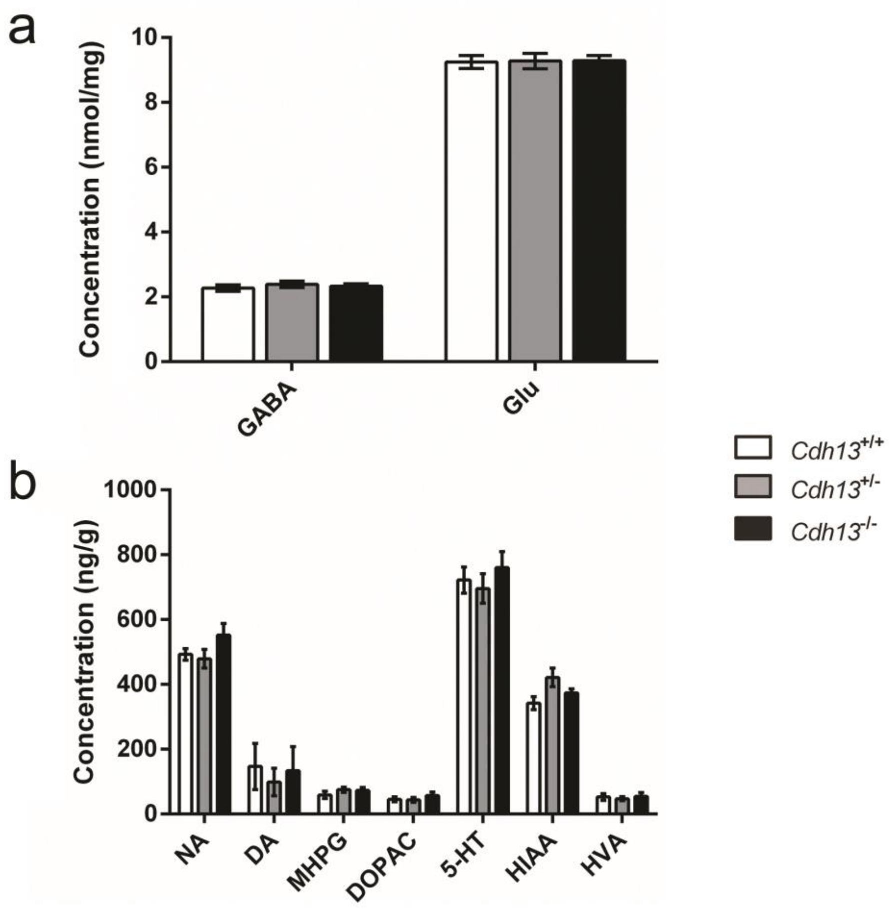

Figure S7. No differences in the concentrations of the most important neurotransmitters and some of their metabolites were found in total hippocampal samples of *Cdh13*$^{+/+}$, *Cdh13*$^{+/-}$ and *Cdh13*$^{-/-}$ mice. (a) Levels of GABA and Glutamate. **(b)** Concentration of monoamines and their metabolites. Data are presented as mean±SEM; *n*=7 per group. Concentrations were statistically compared between genotypes using Kruskal-Wallis test. Abbreviations: Glu, glutamate; GABA, gamma-butyric acid; NA, norepinephrine; DA, dopamine; MHPG, 3-methoxy-4-hydroxyphenylglycol; DOPAC, 3,4-dihydroxyphenylacetic acid; 5-HT, serotonin; 5-HIAA, 5-hydroxy indole acetic acid; HVA, homovanillic acid.

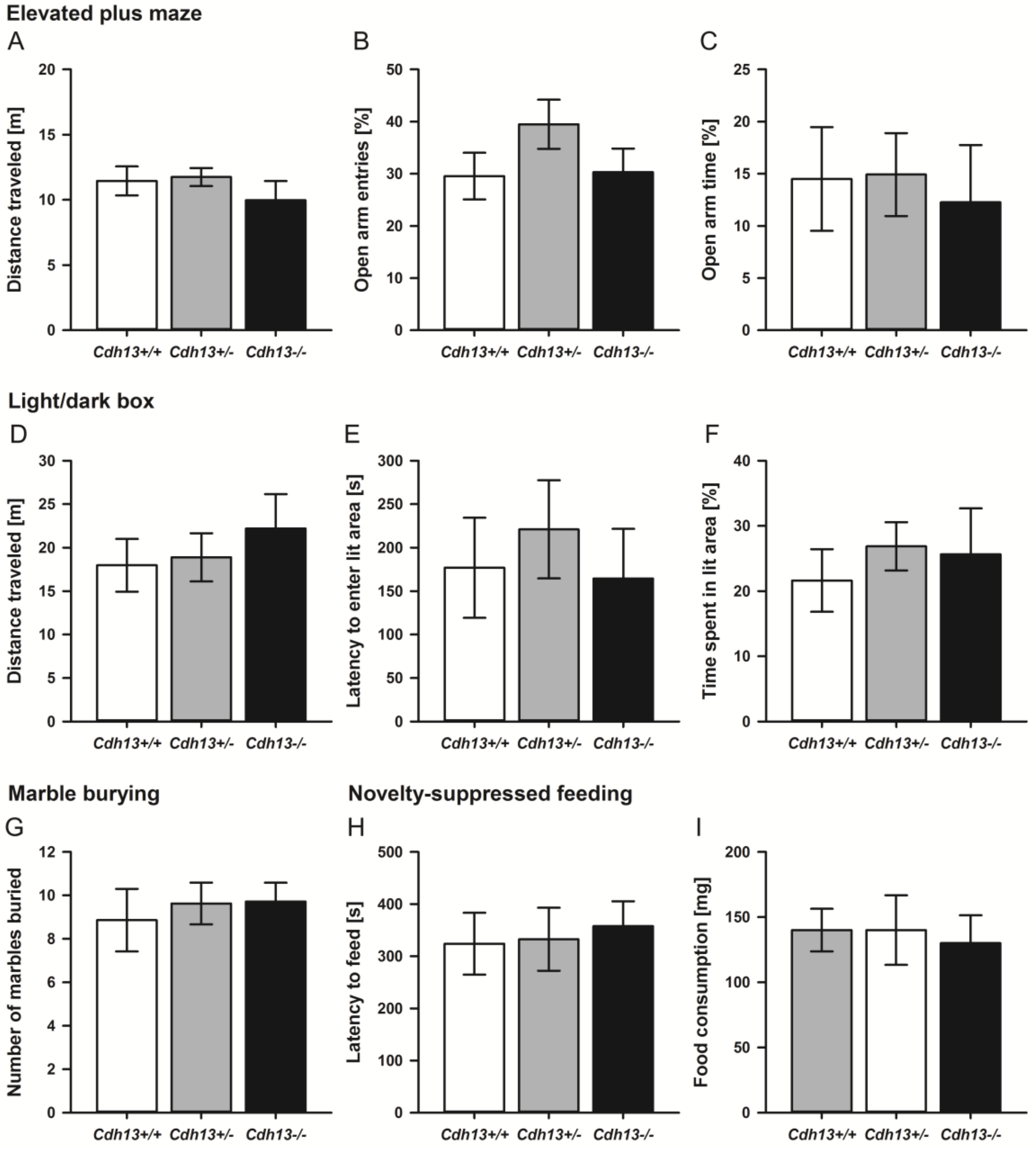

Figure S8. Anxiety-like behavior is not affected by *Cdh13* genotype. (A) Total distance traveled, **(B)** % number of open arm entries and **(C)** % time spent on the open arms of the elevated plus maze did not significantly differ between *Cdh13*$^{+/+}$, *Cdh13*$^{+/-}$ and *Cdh13*$^{-/-}$ mice (*n*=7-8/genotype). **(D)** Total distance traveled, **(E)** latency to enter the lit area and **(F)** % time spent in the lit area of the light/dark box were not significantly altered in *Cdh13*$^{-/-}$ and *Cdh13*$^{+/-}$ mice as compared to *Cdh13*$^{+/+}$ mice. **(G)** No genotype differences were found for the number of marbles buried (*n*=7-8/genotype). **(H)** The latency to feed and **(I)** food consumption during 5 min after the novelty-suppressed feeding test revealed no significant differences between *Cdh13*$^{+/+}$, *Cdh13*$^{+/-}$ and *Cdh13*$^{-/-}$ mice (*n*=10/genotype). Data are presented as mean±SEM.

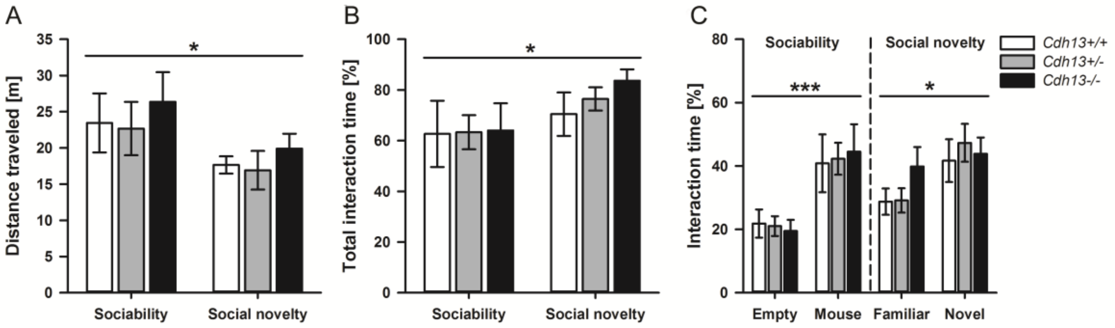

Figure S9. *Cdh13*$^{-/-}$ and *Cdh13*$^{+/-}$ mice show normal social behavior. (A) Total distance traveled was significantly decreased during the test of preference for social novelty as compared to the sociability test. However, no significant genotype effect was observed for the total distance traveled. **(B)** Total interaction time was significantly increased during the test of preference for social novelty as compared to the sociability test. However, no significant genotype effect was observed for total interaction time. (**C**, left panel) In the first trial, all mice showed significant levels of sociability (preference towards the cage containing an unfamiliar mouse over an empty cage). (**C**, right panel) In the second trial, all mice showed preference for social novelty (significantly increased interaction time with the novel mouse compared to the familiar mouse). *N*=7-8/genotype. Data are presented as mean±SEM. *$p<0.05$, ***$p<0.001$.

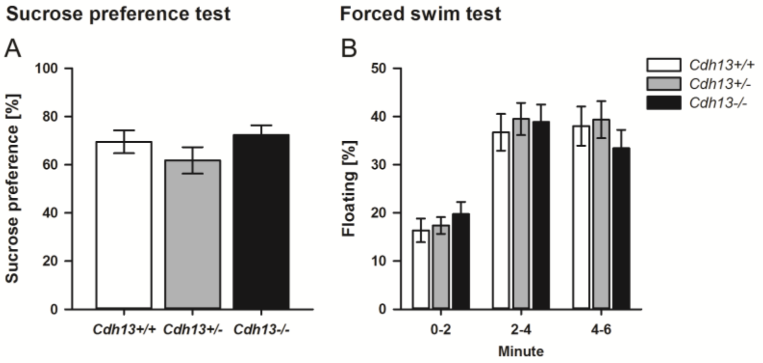

Figure S10. Depression-like behavior is not affected by *Cdh13* genotype. (A) Sucrose preference, a measure of anhedonia, was not significantly altered in *Cdh13*$^{-/-}$ and *Cdh13*$^{+/-}$ mice compared to *Cdh13*$^{+/+}$ mice (*n*=6-7 cages/genotype, 2 mice/cage). **(B)** Floating duration in the Porsolt swim test, a measure of behavioral despair, was similar in all three genotypes (*n*=17-20/genotype). Data are shown as mean±SEM.

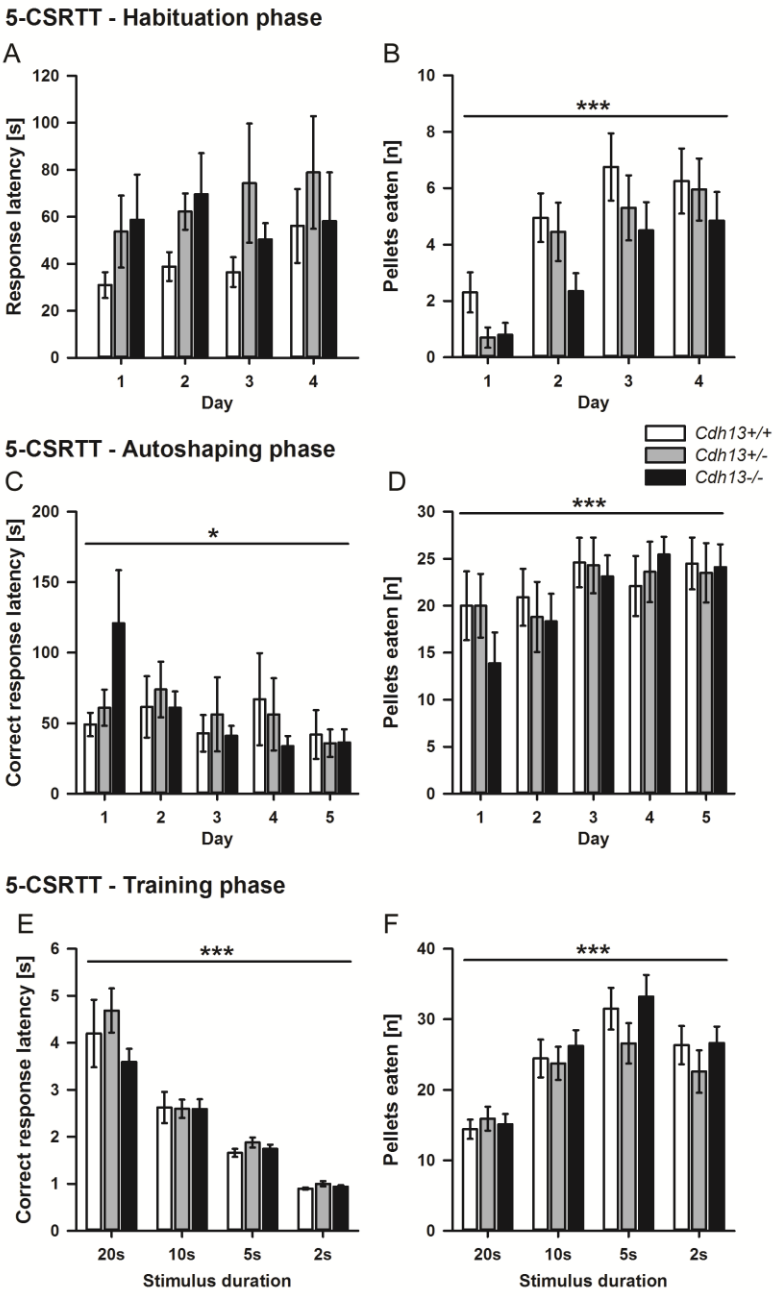

Figure S11. *Cdh13*$^{-/-}$ and *Cdh13*$^{+/-}$ mice show normal habituation, autoshaping and training performance in the 5-CSRTT. (A) The average response latency did not significantly differ between genotypes and habituation sessions. **(B)** The number of pellets eaten significantly increased over habituation sessions in all mice. **(C)** The average latency to make a correct response significantly decreased and **(D)** the number of pellets eaten increased in all genotypes over autoshaping sessions. **(E)** The latency to make a correct response significantly decreased and **(F)** the number of pellets eaten significantly increased in all mice when the stimulus duration decreased. *N*=10/genotype. Data are shown as mean±SEM. *$p<0.05$, ***$p<0.001$.

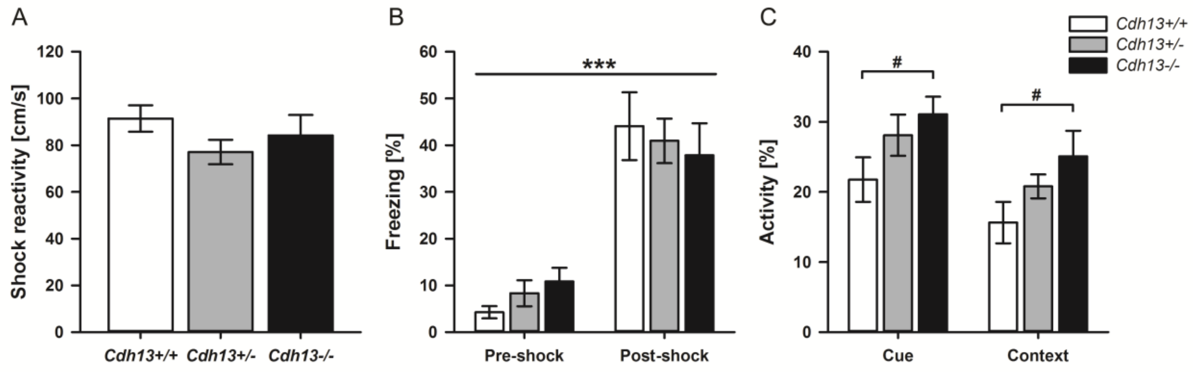

Figure S12. Footshock reactivity and acquisition of conditioned fear is not affected by *Cdh13* genotype. (A) Reactivity to footshock, as measured by the maximum velocity, did not significantly differ between *Cdh13*$^{+/+}$, *Cdh13*$^{+/-}$ and *Cdh13*$^{-/-}$ mice. **(B)** Pre-shock freezing (before the first CS-US pairing) and post-shock freezing (after the last CS-US pairing) was similar in all three genotypes. However, post-shock freezing was significantly higher than pre-shock freezing, indicating normal acquisition of conditioned fear in all mice. **(C)** Locomotor activity tended to be higher in *Cdh13*$^{-/-}$ mice compared to *Cdh13*$^{+/+}$ mice during both cued and contextual fear memory test. N=9-10/genotype. Data are shown as mean±SEM. $^{\#}p<0.1$, $^{***}p<0.001$.

SUPPLEMENTARY REFERENCES

1. Schwenk F, Baron U, Rajewsky K. A cre-transgenic mouse strain for the ubiquitous deletion of loxP-flanked gene segments including deletion in germ cells. *Nucleic Acids Res* 1995; **23**: 5080-5081.

2. Skarnes WC, Rosen B, West AP, Koutsourakis M, Bushell W, Iyer V *et al.* A conditional knockout resource for the genome-wide study of mouse gene function. *Nature* 2011; **474**: 337-342.

3. Rodriguez CI, Buchholz F, Galloway J, Sequerra R, Kasper J, Ayala R *et al.* High-efficiency deleter mice show that FLPe is an alternative to Cre-loxP. *Nat Genet* 2000; **25**: 139-140.

4. Riederer PF, Burger R. Ist Schokolade ein Psychopharmakon? Die Rolle von ß-Phenylethylamin als Psychostimulus. *Psychopharmakotherapie* 2009; **16**: 26-31.

5. Bonn M, Schmitt A, Asan E. Double and triple in situ hybridization for coexpression studies: combined fluorescent and chromogenic detection of neuropeptide Y (NPY) and serotonin receptor subtype mRNAs expressed at different abundance levels. *Histochem Cell Biol* 2012; **137**: 11-24.

6. Watakabe A, Ohsawa S, Hashikawa T, Yamamori T. Binding and complementary expression patterns of semaphorin 3E and plexin D1 in the mature neocortices of mice and monkeys. *J Comp Neurol* 2006; **499**: 258-273.

7. Karabeg MM, Grauthoff S, Kollert SY, Weidner M, Heiming RS, Jansen F *et al.* 5-HTT deficiency affects neuroplasticity and increases stress sensitivity resulting in altered spatial learning performance in the Morris water maze but not in the Barnes maze. *PLoS One* 2013; **8**: e78238.

8. Paxinos G, Franklin KBJ. *The mouse brain in stereotaxic coordinates*. Elsevier Academic Press: San Diego, CA, 1997.

9. Guzman SJ, Schlogl A, Schmidt-Hieber C. Stimfit: quantifying electrophysiological data with Python. *Front Neuroinform* 2014; **8**: 16.

10. Tricoire L, Pelkey KA, Erkkila BE, Jeffries BW, Yuan X, McBain CJ. A blueprint for the spatiotemporal origins of mouse hippocampal interneuron diversity. *J Neurosci* 2011; **31**: 10948-10970.

11. Marx M, Gunter RH, Hucko W, Radnikow G, Feldmeyer D. Improved biocytin labeling and neuronal 3D reconstruction. *Nat Protoc* 2012; **7**: 394-407.

12. Belzung C, Griebel G. Measuring normal and pathological anxiety-like behaviour in mice: a review. *Behav Brain Res* 2001; **125**: 141-149.

13. Pellow S, File SE. Anxiolytic and anxiogenic drug effects on exploratory activity in an elevated plus-maze: a novel test of anxiety in the rat. *Pharmacol Biochem Behav* 1986; **24**: 525-529.

14. Post AM, Weyers P, Holzer P, Painsipp E, Pauli P, Wultsch T *et al.* Gene-environment interaction influences anxiety-like behavior in ethologically based mouse models. *Behav Brain Res* 2011; **218**: 99-105.

15. Dulawa SC, Hen R. Recent advances in animal models of chronic antidepressant effects: the novelty-induced hypophagia test. *Neurosci Biobehav Rev* 2005; **29**: 771-783.

16. van Gaalen MM, Stenzel-Poore M, Holsboer F, Steckler T. Reduced attention in mice overproducing corticotropin-releasing hormone. *Behav Brain Res* 2003; **142**: 69-79.

17. Holter SM, Kallnik M, Wurst W, Marsicano G, Lutz B, Wotjak CT. Cannabinoid CB1 receptor is dispensable for memory extinction in an appetitively-motivated learning task. *Eur J Pharmacol* 2005; **510**: 69-74.

18. Touma C, Palme R, Sachser N. Analyzing corticosterone metabolites in fecal samples of mice: a noninvasive technique to monitor stress hormones. *Horm Behav* 2004; **45**: 10-22.
